# Bridging the Gap: The Importance of TUBA1A α-Tubulin in Forming Midline Commissures

**DOI:** 10.3389/fcell.2021.789438

**Published:** 2022-01-19

**Authors:** Georgia Buscaglia, Kyle R. Northington, Jayne Aiken, Katelyn J. Hoff, Emily A. Bates

**Affiliations:** ^1^ Department of Pediatrics, University of Colorado Anschutz Medical Campus, Aurora, CO, United States; ^2^ Department of Cell and Developmental Biology, University of Colorado Anschutz Medical Campus, Aurora, CO, United States

**Keywords:** TUBA1A protein, microtubule cytoskeleton, MAP1B, commissure defects, tubulinopathy

## Abstract

Developing neurons undergo dramatic morphological changes to appropriately migrate and extend axons to make synaptic connections. The microtubule cytoskeleton, made of α/β-tubulin dimers, drives neurite outgrowth, promotes neuronal growth cone responses, and facilitates intracellular transport of critical cargoes during neurodevelopment. *TUBA1A* constitutes the majority of α-tubulin in the developing brain and mutations to *TUBA1A* in humans cause severe brain malformations accompanied by varying neurological defects, collectively termed tubulinopathies. Studies of *TUBA1A* function in mammalian cells have been limited by the presence of multiple genes encoding highly similar tubulin proteins, which leads to α-tubulin antibody promiscuity and makes genetic manipulation challenging. Here, we test mutant tubulin levels and assembly activity and analyze the impact of TUBA1A reduction on growth cone composition, neurite extension, and commissural axon architecture during brain development. We present a novel tagging method for studying and manipulating TUBA1A in cells without impairing tubulin function. Using this tool, we show that a *TUBA1A* loss-of-function mutation *TUBA1A* ^*N102D*^ (*TUBA1A* ^*ND*^), reduces TUBA1A protein levels and prevents incorporation of TUBA1A into microtubule polymers. Reduced Tuba1a α-tubulin in heterozygous *Tuba1a* ^*ND/+*^ mice leads to grossly normal brain formation except a significant impact on axon extension and impaired formation of forebrain commissures. Neurons with reduced Tuba1a as a result of the *Tuba1a* ^*ND*^ mutation exhibit slower neuron outgrowth compared to controls. Neurons deficient in Tuba1a failed to localize microtubule associated protein-1b (Map1b) to the developing growth cone, likely impacting stabilization of microtubules. Overall, we show that reduced Tuba1a is sufficient to support neuronal migration and cortex development but not commissure formation, and provide mechanistic insight as to how *TUBA1A* tunes microtubule function to support neurodevelopment.

## Introduction

Mammalian brain development is a complex process that requires precise coordination of multiple cell types and extracellular cues to form a fully specified tissue. Despite many advances in understanding the cellular and molecular players involved in brain development, there is still much that remains unknown. Insights into the molecular pathways governing neurodevelopment can be gained from studying genetic mutations that impair specific aspects of brain development. Severe cortical and neurodevelopmental phenotypes associated with mutations that disrupt tubulin genes, termed tubulinopathies, have recently been described in humans (Poirier et al., 2007; Fallet-Bianco et al., 2008; Cushion et al., 2013; Oegema et al., 2015). Tubulinopathy mutations cause a spectrum of neurodevelopmental phenotypes, but frequently involve cortical malformations such as lissencephaly, agenesis or hypoplasia of the corpus callosum, and cerebellar hypoplasia (Poirier et al., 2007; Tischfield et al., 2011; Cushion et al., 2013; Oegema et al., 2015). Recent studies of human tubulinopathy mutations have revealed that each variant may impact different aspects of microtubule function, such as protein folding, polymerization competency, and microtubule-associated protein (MAP)-binding (Tian et al., 2008; Tian et al., 2010; Aiken et al., 2019a; Aiken et al., 2019b). Tubulin mutations can therefore be used to interrogate the requirement for specific aspects of microtubule function throughout neurodevelopment.

Developing neurons must migrate to the correct location, extend axons to meet sometimes distant synaptic partners, and form functional connections. Throughout this process, neurons undergo dramatic morphological changes that require coordinated interaction between the cytoskeleton and the extracellular environment. In post-mitotic neurons, microtubule polymers made of α/β-tubulin dimers facilitate nucleokinesis and cellular migration, support growth cone navigation, promote axon formation and form the tracks upon which intracellular trafficking occurs (Sakakibara et al., 2013; Liu and Dwyer, 2014; Kapitein and Hoogenraad, 2015; Miller and Suter, 2018). The microtubule network needs to be precisely controlled to fulfill diverse functions in neurons. Microtubule properties can be modulated through post-translational modifications (PTMs) to tubulin subunits, association with MAPs, and through the particular tubulin genes, or isotypes, that a cell expresses (Gadadhar et al., 2017). The human genome contains at least nine unique α- and ten unique β-tubulin genes (Khodiyar et al., 2007; Findeisen et al., 2014). The α-tubulin isotype encoded by the gene *TUBA1A* is abundant in the brain and is the most highly expressed α-tubulin in post-mitotic developing neurons (Miller et al., 1987; Gloster et al., 1994; Gloster et al., 1999). *TUBA1A* mutations are highly represented in cases of human tubulinopathies (Aiken et al., 2017), suggesting that *TUBA1A* plays an important role in neurodevelopment. However, the high degree of sequence conservation between α-tubulin genes has historically made studying *TUBA1A* function in cells challenging, due to the limited availability of tools.

Mouse models harboring mutations to *Tuba1a* can be used as tools to interrogate the function of Tuba1a in the context of the neuronal milieu. As tubulin genes are often required for life and the nucleotide sequence between isotypes is conserved, generation of mutant mouse lines to study *Tuba1a* function *in vivo* has been challenging. To date, only a handful of *Tuba1a* mutant mouse lines have been generated, three by ENU-induced forward genetic screens and one by site-directed CRISPR gene editing (Keays et al., 2007; Gartz Hanson et al., 2016; Bittermann et al., 2019; Leca et al., 2020). We previously revealed that the ENU-induced *Tuba1a* ^*N102D*^ allele (*Tuba1a* ^*ND*^) impairs microtubule function in both *S. cerevisiae* and mice through a loss-of-function mechanism by reducing α-tubulin protein (Gartz Hanson et al., 2016; Buscaglia, 2020). Homozygous *Tuba1a* ^*ND*^ mice exhibit severely impaired brain development and are neonatal lethal, similar to phenotypes seen in the *Tuba1a* ^*null*^ and *Tuba1a*-R215* mutant mice (Gartz Hanson et al., 2016; Aiken et al., 2017; Bittermann et al., 2019). In homozygous *Tuba1a* ^*ND*^, *Tuba1a* ^*Null*^ and *Tuba1a*-R215* mice, as well as many patients with *TUBA1A*-associated tubulinopathies, cortical migration and commissural formation are severely disrupted. This makes it difficult to infer whether axon pathfinding deficits are a direct consequence of altered Tuba1a function or if they are secondary to abnormal cortical layering and migration. *Tuba1a* ^*ND/+*^ heterozygous mutant mice have reduced Tuba1a function during brain development, which is sufficient to support neuron survival and cortical layer formation (Gartz Hanson et al., 2016; Buscaglia, 2020), but does not support formation of axon commissures. Therefore, *Tuba1a* ^*ND/+*^ heterozygous animals can provide insight into how Tuba1a contributes specifically to axon pathfinding.

Here, we show that a reduction in developmental Tuba1a protein impairs formation of large brain commissures. Using a novel tubulin visualization technique, we demonstrate that the *TUBA1A* ^*N102D*^ mutation prevents incorporation of *TUBA1A* into microtubule polymers in cells. In mice, heterozygous *Tuba1a* ^*ND/+*^ brains fail to form the corpus callosum, anterior and hippocampal commissures. Cultured neurons from *Tuba1a* ^*ND/+*^ and wild-type cortices reveal that *Tuba1a* ^*ND/+*^ neurons have shorter neurites than wild-type. Further, we demonstrate that *Tuba1a* ^*ND/+*^ neurons fail to localize Map1b, a critical developmental MAP, to the developing growth cone. Neither Map1b expression nor binding to microtubules is impaired by reduced Tuba1a function. Trafficking along neurites is perturbed by reduced Tuba1a. Thus failure to localize Map1b is likely due to defects in trafficking. Collectively, our data present evidence that reduction of functional Tuba1a protein allows for normal neuron migration and cortical layering but prevents formation of commissures by impairing axon outgrowth.

## Materials and Methods

### Animals

All animal research was performed in accordance with the Institutional Animal Care and Use Committee at the University of Colorado School of Medicine. All mice used were maintained on a 129S1/C57Bl6 genetic background. Mice were kept on a 12:12 light:dark cycle with *ad libitum* access to food and water. *Tuba1a* ^*ND/+*^ mice were identified in an ENU mutagenesis screen that was conducted using C57Bl/6J mice and then outcrossed onto 129S1/Svlmj (Gartz Hanson et al., 2016). All of the data for this manuscript were obtained from mice that have been outcrossed over 20 generations to 129S1. *Tuba1a* ^*ND/+*^ and wild-type littermate mice were maintained on water supplemented with 0.2 g/L MgSO_4_ to promote *Tuba1a* ^*ND/+*^ survival and ability to reproduce. Survival rates of *Tuba1a* ^*ND/+*^ with 0.2 g/L MgSO_4_ are 34/35 total and without MgSO_4_ supplemented water are three out of nine pups. In contrast, 9/10 wild type litter mates survived without MgSO_4_ supplemented water and 47/47 wildtype littermates survive with MgSO_4_ supplemented water. For timed matings, male and female mice (*Tuba1a* ^*ND/+*^ x wild type) were introduced overnight and separated upon confirmation of mating, which was considered embryonic day 0.5. Male and female mice were represented in all studies. All mice were genotyped by PCR amplification of DNA isolated from tail snips followed by Sanger sequencing to differentiate homozygous or heterozygous *Tuba1a* ^*ND/+*^ mice from wild-type. Primers used to amplify mouse DNA for genotyping were: forward:TGGATGGTACGCTTGGTCTT; reverse: CTT​TGC​AGA​TGA​AGT​TCG​CA; and sequencing: GTC​GAG​GTT​TCT​ACG​ACA​GAT​ATC.

### Histology

Mice were anesthetized and *trans*-cardially perfused with 0.1 M NaCl and 4% paraformaldehyde (PFA) for histology. Tissues of interest were dissected and post-fixed in 4% PFA. Tissue sectioning was performed on a CM1520 cryostat (Leica, Wetzlar, Germany) and 30 μm cryosections were obtained for all histology. For luxol fast blue staining, sections from brain were stained using a 0.1% luxol fast blue solution. For most immunofluorescence studies PFA-fixed tissues or cells were blocked in phosphate-buffered saline (PBS) containing 5% goat serum or bovine serum albumin (BSA) with 0.3% Triton-X 100. Primary and secondary antibodies were diluted in PBS containing 1% BSA with 0.1% Triton-X 100.

### Electron Microscopy

Mice used for electron microscopy were perfused with 0.1 M NaCl and 2.5% glutaraldehyde 4% PFA, after which the brain was dissected and post-fixed in 2.5% glutaraldehyde 4% PFA overnight at 4°C. Following post-fixation, brains were sent for sectioning and imaging by the CU School of Medicine Electron Microscopy Core facility. For analysis, Axons were counted in EM images only if they were captured completely in cross-section (round) and contained either a dark myelin ring surrounding, or had features of an axon (intracellular space had uniform electron density). Structures were only considered as compacted myelin sheaths if they were surrounding a structure resembling a cross-sectional axon and were electron dense.

### Plasmids and Reagents

The hexahisitidine (His6) epitope tag was inserted in the α-tubulin internal loop region (Schatz et al., 1987; Heilemann et al., 2008; Hotta et al., 2016). Codon optimization for *rattus norvegicus* (https://www.idtdna.com/codonopt) was used to generate the His6 sequence CAT​CAC​CAC​CAT​CAT​CAC, which was inserted into the coding region of human *TUBA1A* from the Mammalian Genome Collection (clone ID:LIFESEQ6302156) between residues I42 and G43. Gibson cloning was used to insert the gBlock of *TUBA1A* internally tagged with His6 (*TUBA1A*-His6) into the pCIG2 plasmid (shared by Dr. Matthew Kennedy, University of Colorado) linearized with NruI and HindIII. *TUBA1A*-His6 incorporation was confirmed by sequencing across the complete *TUBA1A* coding region. The *TUBA1A* ^*T349E*^ (*TUBA1A* ^*TE*^) polymerization incompetent, and *TUBA1A* ^*E255A*^ (*TUBA1A* ^*EA*^) highly polymer-stable α-tubulin mutants were identified and described in prior publications (Anders and Botstein, 2001; Johnson et al., 2011; Roostalu et al., 2020). To generate the GFP-TUBA1A vector, the coding region of human TUBA1A from the Mammalian Genome Collection (clone ID:LIFESEQ6302156) was amplified with forward primer TAT​GGC​GGC​CGC​AGA​GTG​CTG​GTA​GTG​CTG​GTA​GTG​CTG​GTA​TGC​GTG​AGT​GCA​TCT​CC and reverse primer TAT​GGC​GGC​CGC​TTA​GTA​TTC​CTC​TCC​TTC​TTC​CTC​ACC. The resulting amplicon encompasses the BsrGI cutsite present at the end of GFP, a linker sequence (tripeptide linker SAG), the TUBA1A coding region, and terminates in a NotI site. This amplicon was cloned into the pCIG2 vector at the end of the GFP sequence using sticky-end cloning with BsrGI and NotI. The GFP-MACF43 vector was shared by Dr. Laura Anne Lowery (Boston College) and Dr. Casper Hoogenraad (Utrecht University). Myr-TdTomato plasmid DNA was shared from Dr. Mark Gutierrez and Dr. Santos Franco (University of Colorado).

### Cell Culture and Nucleofection

Cos-7 cells (Thermo Fisher Scientific, Waltham, MA; ATCC^®^ CRL-1651™) were cultured in a 37°C humidified incubator with 5% CO_2_ in DMEM (Gibco) supplemented with 10% fetal bovine serum (Gibco), 1 mM sodium pyruvate (Thermo), and penicillin/streptomycin (1,000 IU/1,000 μg/ml; Thermo). Cos-7 cells were transfected with 2.5 µg of hexahistidine (His6) tagged *TUBA1A* plasmid DNA using Lipofectamine 3000 (Invitrogen). The Cos-7 cells were fixed and imaged 24 h after the addition of the *TUBA1A* plasmid. Cells were washed with PBS and PHEM buffer (60 mM PIPES, 25 mM HEPES, 10 mM EGTA, 2 mM Mg2Cl), and then fixed in 2% formaldehyde plus 0.05% glutaraldehyde. Immunostaining was performed using primary antibodies directed against: 6X-Histidine (Invitrogen, 4A12E4 37-2900; 1:500), Acetylated Tubulin (Cell Signaling Technology, D20G3; 1:800). Primary antibodies were diluted in blocking buffer (5% BSA with 0.3% Triton-X 100 in PBS) and incubated overnight at 4°C in a humidified chamber. After primary antibody staining, cells were washed three times with PBS. Fluorescently-conjugated secondary antibodies were diluted 1:500 in 1% BSA with 0.1% Triton-X 100 in PBS and incubated for 1 h at room temperature, protected from light. Secondary antibodies were from Life Technologies (Carlsbad, CA) all used at 1:500. For Cos-7 proteasome inhibition assays, 5 µM Lactacystin A (Tomoda and Omura, 2000; Ōmura and Crump, 2019) was added to normal culture medium for 24 h, the day following transfection with *TUBA1A*-His6 constructs. Dissociated neurons were cultured from male and female P0-P2 mouse or rat cortices. Brains were removed and placed into Hanks Balanced Salt Solution (HBSS, Life Technologies) supplemented with 9.9 mM HEPES (Life Technologies) and 1 mM kynurenic acid (Tocris Bioscience, Bristol, United Kingdom). Meninges were removed and cortices were dissected and cut into approximately 1 mm pieces. Cortical pieces were triturated to a single-cell suspension using glass Pasteur pipettes. Cortical neurons were plated onto 35 mm Poly-D-Lysine coated glass-bottom culture dishes at a density of 350,000 cells (Willco Wells, HBSt-3522). For nucelofected mouse and rat neurons, 4 µg of plasmid DNA was introduced to 4 × 10^6^ neurons using an AMAXA nucleofection kit (VPG-1001, VPG-1003; Lonza). AMAXA-nucleofected cells were plated in 35 mm glass bottom imaging dishes. Neurons were maintained in a 37°C humidified incubator with 5% CO_2_ in phenol-free Neurobasal-A medium (Life Technologies) supplemented with B-27 (Thermo Fisher Scientific, Waltham, MA), Penn/strep (Thermo), GlutaMax (Thermo), 5 ng/ml β-FGF (Gibco), and Sodium Pyruvate (Thermo).

### RNA Isolation + RTPCR

RNA was isolated from Cos-7 cells, 48 h post-transfection using TRIzol Reagent (Thermo; 15596026). RNA concentration and purity were determined using a spectrophotometer, then cDNA was synthesized using the RT2 First Strand Kit (Qiagen, Hilden, Germany; 330401). qRT-PCR reactions were prepared with SYBR Green RT-PCR Master mix (Thermo; S9194) and run with a CFX Connect Real-Time System (Bio-Rad). Samples were run in triplicate, results were analyzed in Excel. All qPCR data presented in this manuscript was normalized to expression of GFP, which was present on the same plasmid as *TUBA1A*-His6 constructs. Wild-type *TUBA1A* mRNA quantity was set to = 1 and *TUBA1A* ^*ND*^ relative mRNA quantity was presented relative to wild-type. For all qRT-PCR experiments 3 biological replicates were used per genotype.

### Neuron Immunocytochemistry

DIV 2 primary cortical neurons were washed with PBS and fixed with a fixation solution of 4% PFA and 0.2% glutaraldehyde in PBS for 15 min at room temperature. For tubulin extraction, cells were washed with PBS followed by PHEM buffer (60 mM PIPES, 25 mM HEPES, 10 mM EGTA, 2 mM Mg2Cl) then soluble tubulin dimers were extracted using 0.1% triton with 10 µM taxol and 0.1% DMSO in PHEM buffer. Extracted cells were fixed with 2% PFA and 0.05% glutaraldehyde in PBS for 10 min, washed with PBS and then reduced in 0.1% NaBH4 in PBS for 7 min at room temperature. Cells were then washed with PBS and blocked in 3% BSA and 0.2% Triton in PBS for 20 min at room temperature, with agitation. Immunostaining was performed using primary antibodies directed against: 6X-Histidine (Invitrogen, 4A12E4 37-2900; 1:500), total α-tubulin (Sigma, DM1A T6199; 1:5,000), Acetylated Tubulin (Sigma, T7451; 1:1,000), Tyrosinated Tubulin (Chemicon, MAB 1864; 1:1,000), Map1b (Santa Cruz Biotech, sc-135978; 1:500), Map2 (Novus Biologicals, NB300-213; 1:2,000). Primary antibodies were diluted in blocking buffer (5% BSA with 0.3% Triton-X 100 in PBS) and incubated overnight at 4°C in a humidified chamber. After primary antibody staining, cells were washed three times with PBS. Fluorescently-conjugated secondary antibodies were diluted 1:500 in 1% BSA with 0.1% Triton-X 100 in PBS and incubated for 2 h at room temperature, protected from light. Secondary antibodies were from Life Technologies (Carlsbad, CA) all used at 1:500. Alexa Fluor 568-conjugated Phalloidin (Thermo, A12380; 1:20) was added during secondary antibody incubation for labeling of filamentous actin. Tissues or cells of interest were mounted onto glass microscope slides and sealed with glass coverslips and aqueous mounting media containing DAPI (Vector Laboratories, #H-1200) and imaged on a Zeiss 780 or 880 confocal microscope with a ×40 or ×63 oil objective.

### Western Blotting

Protein was isolated from brains of P0-P2 mice by dounce homogenization and ultra-centrifugation at 100,000 × g for 45 min at 4°C. Map1b was quantified in three different scenarios. Total Map1b was quantified from the whole brain lysates. For Map1b associated with tubulin, the tubulin-enriched fraction was isolated either with taxol at 37°C according to a previously established protocol (Vallee, 1982) or without taxol at 37°C using centrifugation to separate the tubulin-rich fraction. Cos-7 cell protein was extracted using a Tris-Triton lysis buffer with protease inhibitor cocktail (Sigma). Protein concentrations were assessed using a BCA assay (Thermo), and relative concentration was determined using a Synergy H1 microplate reader (BioTek Instruments, Winooski, VT). 5 µg of either whole brain lysate or tubulin-enriched protein fraction was loaded per lane and run on 4–20% Mini-Protean TGX Stain-Free precast gels (4568093; Bio-Rad Laboratories, Hercules, CA) at 150 mV for 1 h. Prior to protein transfer, Stain-Free gels were activated with UV light for 1 min and imaged for total protein on gel using a ChemiDoc MP imager (Bio-Rad). Proteins were transferred to PVDF blotting membranes (Bio-Rad) in standard 25 mM Tris-base, 192 mM glycine, 15% methanol transfer buffer, or transfer buffer optimized for high molecular-weight proteins (>200 kDa) by the addition of 0.025% SDS. Blots were transferred at 4°C and 75 V for either 1 h for standard molecular-weight proteins, or 3 h for high molecular-weight proteins. Immediately following transfer, PVDF membranes were rinsed in TBST and imaged to quantify the total protein on blot using UV-activated Stain-Free blots. Gels were also imaged post-transfer to assess transfer efficiency for each blot. Membranes were blocked in Tris-buffered Saline containing 0.1% Tween-20 (TBST) with 5% BSA for 1 h and incubated in primary antibody diluted in TBST containing 1% BSA overnight at 4°C. Primary antibodies were: mouse anti 6X-Histidine (Invitrogen, 4A12E4; 1:500), chicken anti-GFP (Invitrogen, A10262; 1:1,000), and mouse anti-Map1b (Santa Cruz, sc-135978; 1:500). Blots were incubated in HRP-conjugated Goat-anti-mouse (1:5,000; Santa Cruz) or goat-anti-chicken (1:5,000; Santa Cruz) secondary antibody diluted in TBST containing 0.5% BSA with streptavidin-HRP (Bio-Rad, 1:10,000) for band visualization for 1 h at room temperature. Blots were developed in ECL solution for 5 min at room temperature (Bio-Rad) and imaged.

### Netrin-1 Experiments

Primary wild-type and *Tuba1a* ^*ND/+*^ neonatal (P0-P3) mouse cortical neurons were nucleofected with 2 µg of Myr-TdTomato plasmid DNA and cultured as described above. Netrin-1 expressing plasmid (OriGene Cat#: MG223704) was transfected into Cos-7 cells using Lipofectamine 3000 (Thermo Fisher Scientific Cat#: L3000015). Cells were washed and media was replaced with Opti-MEM (Thermo Fisher Scientific Cat#: 31985062). The following day media was removed from cells and placed in an equilibrated Amicon 30 kDa molecular weight cut-off ultracentrifuge tube (Millipore Sigma Cat#: UFC903008). The purified Netrin-1 was collected and quantified using a BCA assay (Thermo Fisher Scientific Cat #:23227). Neurons were measured at DIV1 at 10 min intervals for 60 min, Netrin-1 was added at a final concentration of 500 ng/ml to the media and images were taken for 60 min after the addition of the chemoattractant. Neurons were measured using ImageJ/FIJI software (NIH) and Excel (Microsoft). Statistical analyses were performed, and graphs were created using Prism version 9.0 (GraphPad). For all statistical analyses, *p* < 0.05 was considered statistically significant. Statistical analyses used in each experiment are indicated in their respective figure legends. T-tests were used for any comparisons between two groups and ANOVA was used for any comparison of more than two groups.

### dSTORM Super-Resolution Imaging and Analysis

Primary neuronal cultures were generated and immunostained as described in the *Cell Culture and Nucleofection* and *Neuron Immunocytochemistry* sections, excepting the mounting step. Instead, an imaging buffer composed of 50 mM Tris (pH 8), 10 mM NaCl, 10% glucose, 10 mM mercaptoethylamin (MEA, Sigma-Aldrich, M6500), 0.56 mg/ml glucose oxidase (Sigma-Aldrich, G0543), and 34 μg/ml catalase (Sigma-Aldrich, C3155) was sterile filtered onto the sample. The cells were then imaged on a Ziess Elyra P.1 microscope using a Plan-Apochromat 63X 1.4NA objective to achieve high excitation power as previously described (Heilemann et al., 2008; Schüttpelz, 2010). In short, the Alexa 647-labeled sample was excited into a high energy state where the majority of fluorophores enter a long-lived dark state while a small fraction of fluorophores are excited into a bright fluorescent state. Over the imaging period, the cycling of fluorophores from the ground state to either of the excited states, stochastically the dark or bright state, provides fluorophore position information. Super-resolution images were generated by running the raw data through a series of analysis steps. First, the fluorescence background was removed using a previously described temporal median filter in Matlab (Matlab code provided by the University of Colorado School of Medicine Advanced Light Microscopy Core) (Hoogendoorn et al., 2014). Fluorophore positions were then fitted using the ThunderSTORM plugin for ImageJ and Fiji (Schneider et al., 2012; Ovesný, 2014). Sample drift was corrected using redundant cross-correlation algorithm in Matlab as previously described (Wang et al., 2014). Finally, a super-resolution image was created by starting out with all pixels set to zero and incrementing the pixel value by one for every molecule localization that falls into that pixel.

### Experimental Design and Statistical Analyses

Band volume of all Western blots was analyzed using Image Lab software (Bio-Rad). Fluorescence intensity measurements, area and morphological assessment, kymograph generation, and quantification of EM images was performed using ImageJ/FIJI software (NIH) and Excel (Microsoft). Statistical analyses were performed, and graphs were created using Prism version 8.0 (GraphPad). Most graphs display all data points to accurately represent the variability in each dataset, except in cases where such presentation obscured visibility. For all statistical analyses, statistical significance was considered to be *p* < 0.05. Statistical analyses used in each experiment are indicated in their respective figure legends. For all graphs mean ± SEM was reported unless otherwise noted. Normality of each dataset was assessed using a Shapiro-Wilk test. In datasets with two groups, parametric data was analyzed using a Student’s t-test, while non-parametric data was assessed by Mann-Whitney *U* analysis of medians. Multiple groups were compared by one-way or two-way ANOVA and analyzed *post hoc* by either a Bonferroni or Kruskal-Wallis test for parametric and non-parametric data, respectively.

## Results

### *TUBA1A* ^*ND*^ α-Tubulin does not Incorporate into Neuronal Microtubules

**FIGURE 1:**
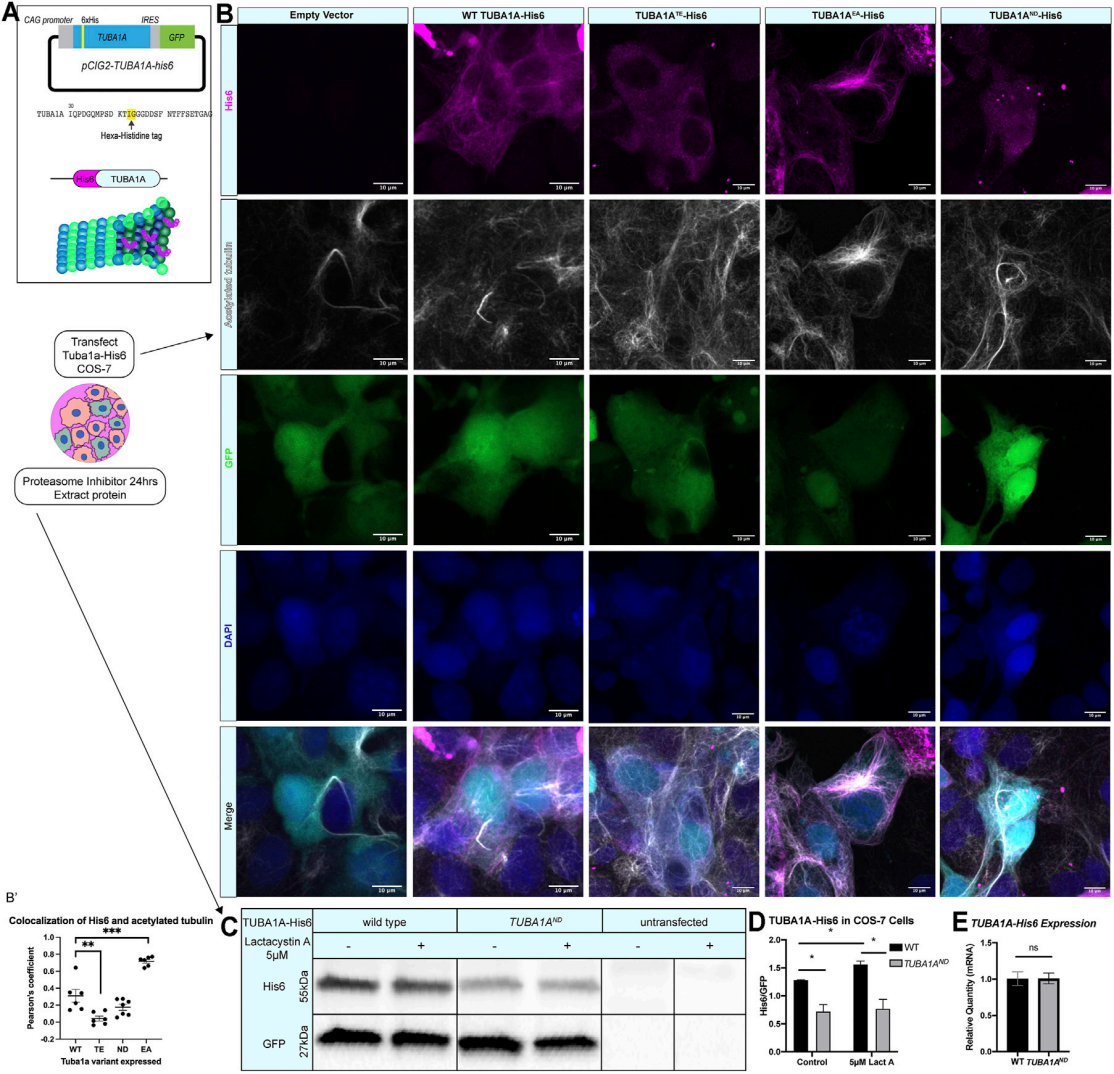
*TUBA1A* ^*ND*^ impairs incorporation into cellular microtubules and reduces α-tubulin protein abundance. **(A)** Schematic of *TUBA1A*-His6 tag and experimental design. His6 epitope tag was added to an internal loop on TUBA1A (top), and TUBA1A-His6 plasmid DNA was transfected into Cos-7 cells to label cellular microtubules in the presence or absence of proteasome inhibitor treatment (bottom). **(B)** Images showing Cos-7 cells transfected with an empty vector control, wild-type *TUBA1A-His6*, *TUBA1A* ^*TE*^ *-His6* (polymerization-incompetent mutant), *TUBA1A* ^*EA*^ *-His6* (highly polymer stable mutant), and *TUBA1A* ^*ND*^ *-His6* plasmid DNA. Cells were immunolabeled with α-His6 antibodies to reveal microtubule incorporation of wild-type and mutant TUBA1A-His6 protein. **(B′)** Scatter plot showing the Pearsons’ correlation coefficient to quantify the co-localization of acetylated tubulin with anti-His6 staining **p < 0.01 and ***p < 0.001. **(C)** Western blot for His6 in TUBA1A-His6 transfected Cos-7 cell lysates. A subset of transfected cells were treated with 5 µM Lactacystin A (LactA) for 24 h to block proteasomal degradation. His6 signal was normalized to GFP, which was expressed from the same plasmid. **(D)** Quantification of band density for His6 western blot shown in **(C)**. His6 band density was normalized to GFP-expressing cells in control-treated (left), and cells treated with 5 µM LactA for 24 h (right). Data were analyzed by *t* test. N = 3 transfections; *n* = 3 technical replicates; *p* = 0.04 for all comparisons marked with asterisks; *p* = 0.83 for control vs. LactA-treated *TUBA1A* ^*ND*^. **(E)** Bar graph representing *TUBA1A* mRNA expression in Cos-7 cells transfected with TUBA1A-His6. *TUBA1A* mRNA expression was normalized to GFP mRNA expression. Data were normalized to *TUBA1A* expression in wild-type (WT) TUBA1A-His6-transfected cells and represent 3 separate transfection experiments and 3 qRT-PCR replicates. Differences between groups were assessed by *t* test (*p* = 0.97). All images were taken at ×40 magnification, scale bars are 10 µm. All graphs show mean of data ± SEM, *p < 0.05.

The high degree of sequence similarity between α-tubulin isotypes has limited study of individual tubulin isotypes at the protein level in cells. As TUBA1A shares 99.5% homology with TUBA1B α-tubulin (only 2 distinct amino acids), commercially available “TUBA1A” specific antibodies are promiscuous and bind more than the intended isotype target. Further, prior attempts to tag TUBA1A neuronal microtubules with N- or C-terminal fluorescent fusion proteins have had detrimental effects on protein function (Gonzalez-Garay and Cabral, 1996; Kimble et al., 2000). These challenges have made the direct visualization of specific tubulin isotypes or mutant tubulin proteins in neurons difficult. Thus, the ways in which TUBA1A specifically contributes to neuronal microtubule protein function have been difficult to ascertain. To address the need for better tools to study TUBA1A protein, we generated a functional hexahistidine (His6)-tagged TUBA1A construct based on a previously identified internal loop in the α-tubulin protein to aid visualization of TUBA1A in mammalian cells (Schatz et al., 1987) (Figure 1A). We inserted the His6 tag into an internal loop of TUBA1A between residues I42 and G43, a region of α-tubulin that tolerates addition of amino acids without disrupting tubulin function (Schatz et al., 1987). Addition of the His6 tag in this loop has previously been used to affinity purify tubulin subunits for reconstituted systems (Heilemann et al., 2008; Hotta et al., 2016). However, to our knowledge this internal His6 tag on α-tubulin has never been used for immunohistochemical assays to visualize isotype-specific localization in cells.

Ectopically expressed wild-type TUBA1A-His6 is incorporated into Cos-7 cell microtubules and colocalizes with polymerized acetylated tubulin (Pearson coefficient = 0.31 ± 0.03, Figures 1B,B′). Along with wild-type TUBA1A-His6, we ectopically expressed three *TUBA1A* variants in Cos-7 cells. Mutant TUBA1A-His6 ectopic expression in cells facilitates evaluation of the abundance and polymerization capability of mutant TUBA1A proteins. *TUBA1A* ^*T349E*^ (*TUBA1A* ^*TE*^) is an α-tubulin mutation that prevents tubulin polymerization in yeast (Johnson et al., 2011) and acts as a negative control. The *TUBA1A* ^*TE*^-His6 shows low levels of punctate His6 staining that does not co-localize with microtubules consistent with previous findings that this mutation prevents TUBA1A^TE^ incorporation into microtubules (Pearson coefficient = 0.042 ± 0.07, Figures 1B,B′). However, acetylated tubulin is clearly visible in TUBA1A^TE^ expressing cells indicating that exogenous expression of TUBA1A^TE^ does not interfere with endogenous microtubule networks. In contrast, *TUBA1A* ^*E255A*^ (*TUBA1A* ^*EA*^) is a microtubule stabilizing α-tubulin mutation which inhibits tubulin depolymerization and thus locks microtubules in a polymer-bound state (Anders and Botstein, 2001; Roostalu et al., 2020) (Figure 1B). The *TUBA1A* ^*EA*^-His6 protein is abundantly incorporated into microtubule polymers (Pearson coefficient 0.72 ± 0.02, Figures 1B,B′). Acetylated tubulin staining in *TUBA1A* ^*EA*^-His6 is also increased compared to wild type. Previous reports demonstrate that the *Tuba1a* ^*ND*^ variant causes partial loss of α-tubulin function in yeast and mice (Gartz Hanson et al., 2016; Buscaglia, 2020), but it was unclear whether or not the ND mutant tubulin is capable of polymerization. Our data reveal that TUBA1A^ND^ -His6 is not visible in microtubule polymers, but can be detected at low levels in the cytoplasm (Pearson coefficient 0.17 ± 0.03, Figures 1B,B′). We conclude that *TUBA1A* ^*ND*^ does not incorporate into microtubules at detectable levels. The acetylated tubulin staining in *TUBA1A* ^*ND*^-His6 cells is comparable to wild-type; microtubules are present, but TUBA1A^ND^ protein is not incorporated.

Western blots of lysates from Cos-7 cells 48 h post-transfection reveal that *TUBA1A* ^*ND*^-His6 protein is significantly reduced compared to wild-type *TUBA1A*-His6 (Figures 1C,D; *p* = 0.04), despite similar *TUBA1A* mRNA levels between wild-type and *TUBA1A* ^*ND*^ transfected Cos-7 cells (Figure 1E; *p* = 0.97). To evaluate if *TUBA1A* ^*ND*^ mutant protein is targeted for proteasomal degradation, we treated *TUBA1A*-His6 transfected Cos-7 cells with 5 µM proteasome inhibitor, Lactacystin A [LactA (Tomoda and Omura, 2000; Ōmura and Crump, 2019)], for 24 h and probed for His6 abundance by western blot (Figure 1C). Treatment with LactA significantly increased wild-type *TUBA1A*-His6 protein compared to control-treated cells, but had no effect on *TUBA1A* ^*ND*^-His6 protein abundance (Figure 1D; *p* = 0.83). These results indicate that *TUBA1A* ^*ND*^ mutant protein is likely not targeted for degradation by the proteasome. Overall, TUBA1A^ND^ mutant protein is not incorporated into microtubules and is less abundant in the cells compared to wild type, despite similar mRNA levels.

**FIGURE 2:**
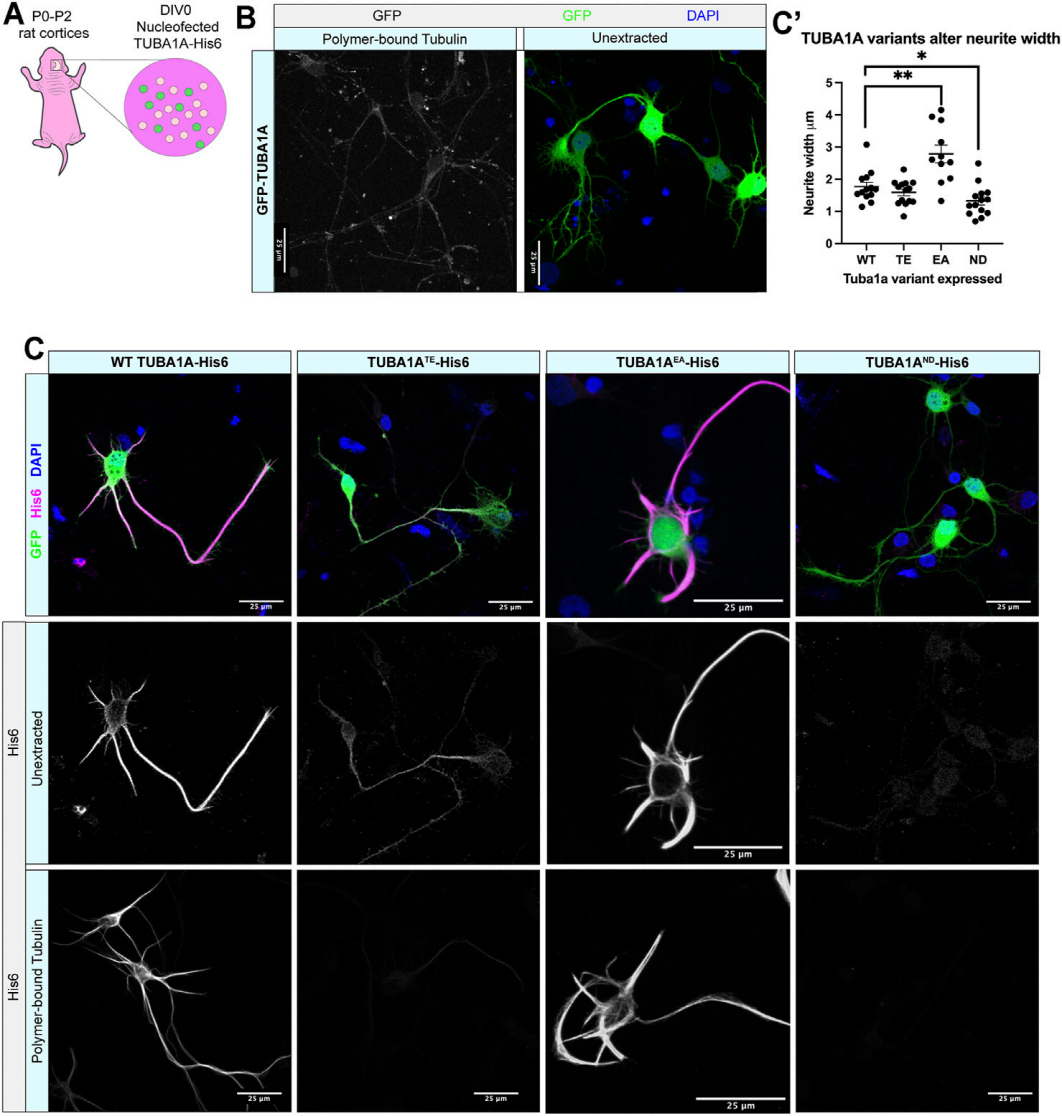
*TUBA1A* ^*ND*^ α-tubulin does not incorporate into neuronal microtubule polymers. **(A)** Schematic of cortical neuron isolation and transfection. **(B)** Cortical rat neurons at DIV 2 transfected with *TUBA1A*-GFP. Left panel shows neurons with soluble tubulin dimers extracted, showing only GFP-labeled TUBA1A that is incorporated into microtubule polymer. Right panel shows neurons with intracellular environment intact (unextracted), containing soluble tubulin dimers and polymerized microtubules transfected to express a TUBA1A-GFP fusion protein. **(C)** Rat cortical neurons at DIV 2 transfected with wild-type (WT) *TUBA1A*-His6 (far left), *TUBA1A* ^*TE*^-His6 polymerization-incompetent mutant as a negative control (middle left), *TUBA1A* ^*EA*^ stabilizing mutant as a positive control (middle right), *TUBA1A* ^*ND*^-His6 (far right). Top panels show composite image containing membrane-bound GFP (green) for confirmation of transfection, α-His6 (Magenta) and DAPI (blue) immunolabeling. Middle panels show unextracted and bottom panels show different DIV2 neurons after tubulin has been extracted to reveal only polymer-bound tubulin, labeled with α-His6 antibodies to visualize ectopic *TUBA1A*-His6 proteins. Scale bar is 25 μm. **(C′)** A Scatter plot shows that neurons expressing His6-Tuba1aEA have significantly wider neurites than neurons expressing His6-Tuba1aWT and neurons expressing His6-Tuba1aND have neurites that are significantly thinner than neurons expressing His6-Tuba1aWT *p < 0.05, **p < 0.01.

We next investigated if *TUBA1A* ^*ND*^ substitution impairs incorporation of TUBA1A protein into neuronal microtubule polymers (Figure 2, Supplementary Figure S1). Wild-type rat cortical neurons were nucleofected with GFP-*TUBA1A*, wild-type *TUBA1A*-His6, *TUBA1A* ^*TE*^-His6 (polymerization incompetent mutant), *TUBA1A* ^*EA*^ *-*His6 (stabilizing mutant)*, and TUBA1A* ^*ND*^-His6 DNA at day *in vitro* 0 (DIV 0; Figure 2A). Following 2 days in culture (DIV 2), cells were fixed and a subset of neurons were permeabilized to remove soluble tubulin dimers (“tubulin extraction”). Extraction of soluble tubulin leaves behind only polymer-bound tubulin, enabling visualization of ectopic tubulin protein polymerization competence (Zieve and Solomon, 1984). Neurons expressing GFP-*TUBA1A* exhibited abundant GFP signal in intact cells, but tubulin extraction revealed that GFP fusion impaired incorporation of TUBA1A into neuronal microtubule polymers (Figure 2B). In contrast, wild-type *TUBA1A*-His6 protein was abundantly visible in both unextracted and extracted neurons, demonstrating that His6-tagging does not impair polymerization ability of TUBA1A in neurons (Figure 2C). As predicted, polymerization incompetent *TUBA1A* ^*TE*^-His6 mutant protein was diffusely visible in intact neurons, but was absent when only polymerized tubulin remained (after extraction) (Figure 2C, Supplementary Figure S1). The microtubule-stabilizing *TUBA1A* ^*EA*^ *-His6* was retained after extraction and incorporated into polymers more abundantly than wild-type, leading to stubby, microtubule-rich neurites (Figures 2C,C′). *TUBA1A* ^*ND*^-His6 protein was detectable at very low levels in unextracted neurons, but was not visible following tubulin extraction, indicating that *TUBA1A* ^*ND*^ impairs incorporation into neuronal microtubules (Figures 2C,C′). These experiments suggest that *TUBA1A* ^*ND*^ reduces abundance of TUBA1A protein and prevents incorporation of mutant TUBA1A into cellular microtubules.

### *Tuba1a* is Required for Formation of Midline Commissures

**FIGURE 3:**
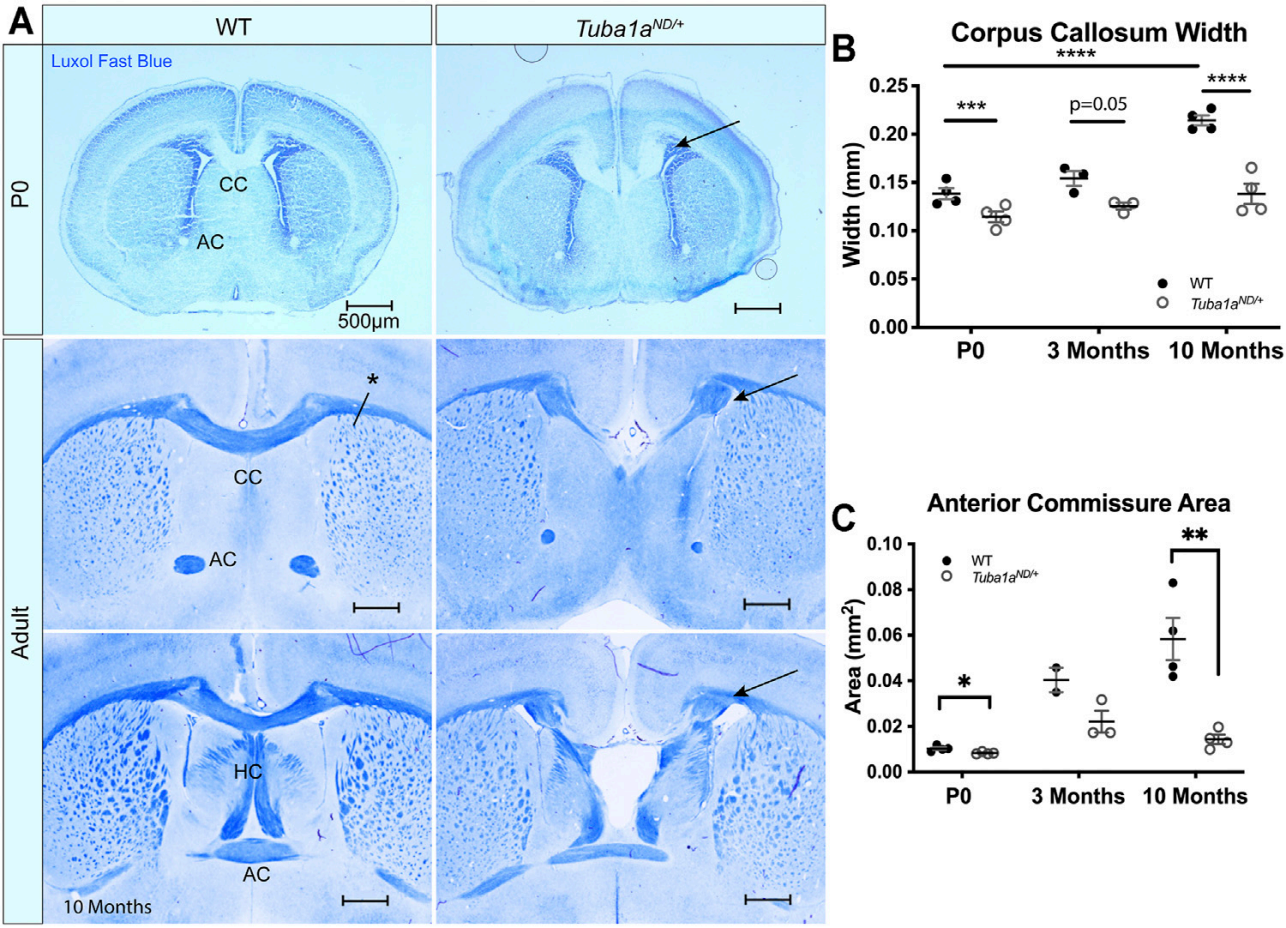
Tuba1a is required for formation of midline commissural structures. **(A)** Luxol fast blue-stained coronal brain sections from postnatal day 0 (P0; top) and Adult (middle-bottom) wild-type and *Tuba1a* ^*ND/+*^ mice. Images portray abnormal midline commissural formation in *Tuba1a* ^*ND/+*^ mouse brains, with labels highlighting the corpus callosum (CC), anterior commissures (AC), and hippocampal commissure (HC). Scale bars are 500 μm. Arrows indicate Probst bundles. Asterisk in A. shows where measurements for B*.* were obtained. **(B)** Scatter plot representing corpus callosum width at P0, 3 months, and 10 months-old. **(C)** Scatter plot displaying anterior commissure area in P0, 3 months, and 10 months-old wild-type and *Tuba1a* ^*ND/+*^ brains. Wild-type and *Tuba1a* ^*ND/+*^ measurements compared by two-way ANOVA. *p < 0.05; **p < 0.01; ***p < 0.001; and ****p < 0.0001.

*TUBA1A* is a major component of developing neuronal microtubules and is critical for proper brain development (Aiken et al., 2017). Human *TUBA1A* tubulinopathy patients with heterozygous mutations in *TUBA1A* exhibit severe brain malformations including defects in commissure formation and changes to cortical folding patterns (lissencephaly, polymicrogyria, pachygyria). While homozygous *Tuba1a* ^*ND/ND*^ mice have severe brain malformations including defects in cortical layer formation (Gartz Hanson et al., 2016), *Tuba1a* ^*ND/+*^ heterozygous mutant mice undergo normal cortical migration, display comparable brain weight to wild-type littermates at birth, and survive to adulthood (Gartz Hanson et al., 2016; Buscaglia, 2020). However, 93% (14/15 brains) have severe defects in corpus collosum formation including 87% (13/15) with complete agenesis of the corpus callosum and abnormal formation of the anterior and hippocampal commissures (Figure 3A). In wild-type mice, 12/12 had well-formed commissures. For a commissure to form properly, nascent callosal “pioneer” axons extend through midline at E15.5, and early “follower” axons begin extending at E17 in mice (Boyer and Gupton, 2018). Evidence of abnormal callosal projections were apparent as early as P0 in *Tuba1a* ^*ND/+*^ brains, as seen by the early formation of aberrant axon bundles adjacent to the callosum, known as Probst bundles (Figure 3A) (Probst, 1901). In addition to agenesis of the corpus callosum at midline, lateral regions of adult *Tuba1a* ^*ND/+*^ corpus callosum were found to be significantly thinner than wild-type (Figure 3B). Similarly, *Tuba1a* ^*ND/+*^ anterior commissures are smaller than that of wild-type littermates (Figure 3C). In wild-type mice, corpus callosum thickness and anterior commissure area both increased significantly between P0 and adulthood; however, normal postnatal expansion of these tracts was not evident in *Tuba1a* ^*ND/+*^ mice (Figures 3B,C). The corpus callosum is absent along the rostral-caudal axis in *Tuba1a* ^*ND/+*^ animals. *Tuba1a* ^*ND/+*^ axons fail to organize into typical midline commissural structures, indicating that half of the normal amount of Tuba1a during brain development is not sufficient for commissural formation (Buscaglia, 2020).

**FIGURE 4:**
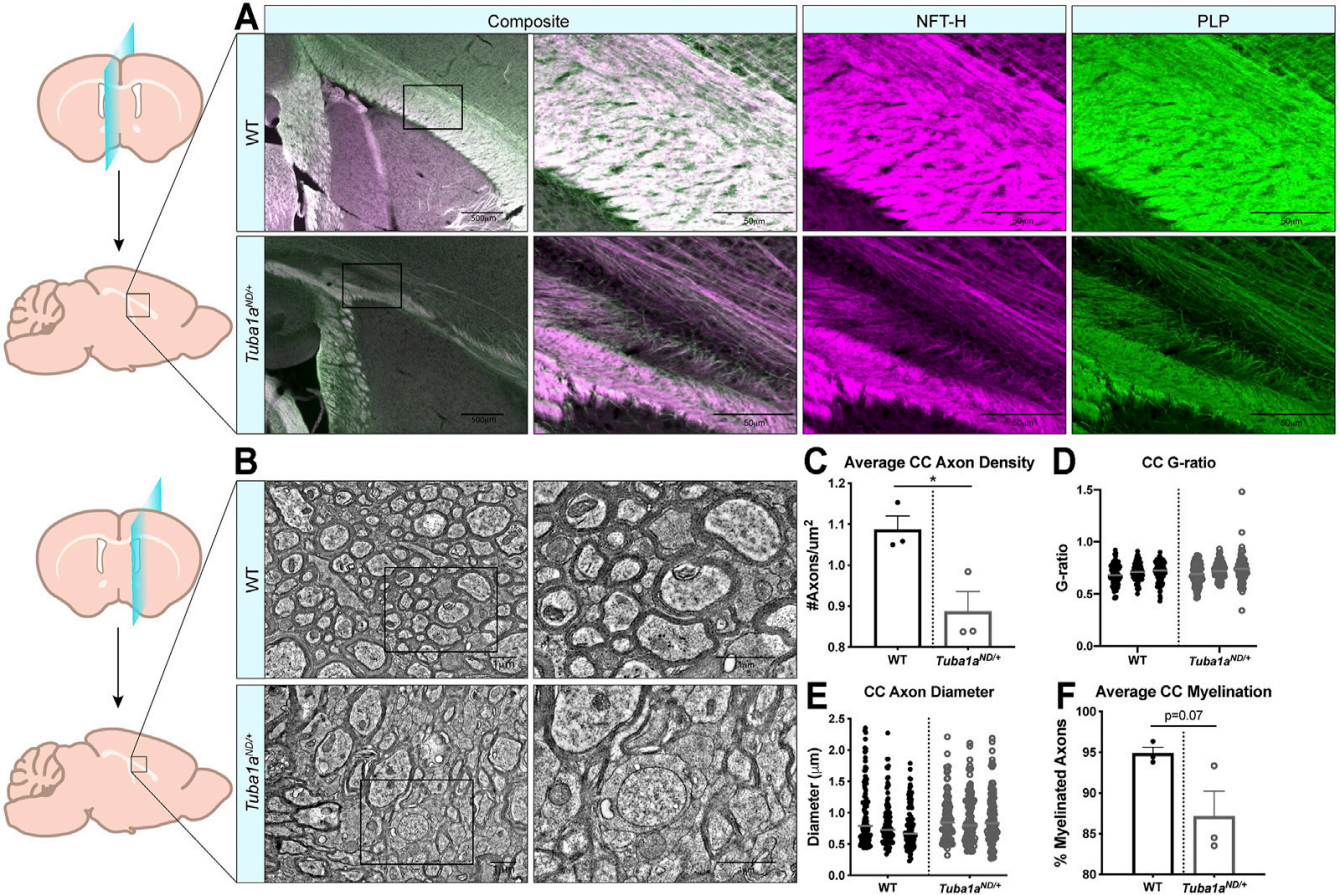
Axon density is reduced by *Tuba1a* ^*ND/+*^ in midline corpus callosum. **(A)** Sagittal brain sections at midline from wild-type (top) and *Tuba1a* ^*ND/+*^ (bottom) brains stained with neurofilament-heavy (NFT-H; magenta) to label axons, and proteolipid protein (PLP; green) to label myelin. Images were taken at ×4 (left) and ×20 (right) magnification with 500 and 50 μm scale bars, respectively. **(B)** Electron microscopy (EM) images of sagittal wild-type (top) and *Tuba1a* ^*ND/+*^ (bottom) sections of corpus callosum 2 mm adjacent to midline. Enlarged regions on right are denoted by boxes, scale bar is 1 μm. **(C)** Scatter plot representing axon density of wild-type and *Tuba1a* ^*ND/+*^ axons in analyzed EM images. Data points represent average values for each animal (*n* = 6 regions containing same total area; *p* = 0.03). **(D)** Scatter plot of G-ratio measurements for wild-type and *Tuba1a* ^*ND/+*^ axons. Data points represent individual myelinated axons and are clustered by animal (N = 3 mice, *n* = 100 axons; *p* = 0.34). **(E)** Scatter plot of axon diameters in wild-type and *Tuba1a* ^*ND/+*^ corpus callosum by EM. Only those axons captured in cross section were assessed for diameter, as skewed axons provide inaccurate measurements. Data points represent individual axons and are clustered by animal (*n* = 100; *p* = 0.14). **(F)** Scatter plot representing the average percent of myelinated axons per animal in wild-type and *Tuba1a* ^*ND/+*^ corpus callosum. Six regions containing the same total area were assessed (*n* = 6; *p* = 0.07). Statistical differences between means of wild-type and *Tuba1a* ^*ND/+*^ datasets were assessed by *t* test, with *p < 0.05.

Examination of sagittal brain sections taken directly at midline revealed dramatic disorganization of corpus callosum axons in *Tuba1a* ^*ND/+*^ brains (Figure 4A). Compared to wild-type, *Tuba1a* ^*ND/+*^ midline commissural axons were largely absent, and the existing axons failed to organize into a tract with uniform orientation (Figure 4A). Despite dramatic differences between wild-type and *Tuba1a* ^*ND/+*^ callosal axon organization, *Tuba1a* ^*ND/+*^ axons were highly colocalized with immunolabeled myelin sheaths (Figure 4A). To further assess the impact of *Tuba1a* ^*ND/+*^ substitution on callosal axon morphology and myelination, we performed electron microscopy (EM) in both wild-type and *Tuba1a* ^*ND/+*^ corpus callosi. Due to the scarcity of axons present directly at midline in the *Tuba1a* ^*ND/+*^ corpus callosum, we sampled a region of corpus callosum 2 mm lateral to midline for both wild-type and *Tuba1a* ^*ND/+*^ animals (Figure 4B). EM images revealed a striking difference in axon density between wild-type and *Tuba1a* ^*ND/+*^ corpus callosi (Figures 4B,C; *p* = 0.03). Myelin thickness, measured by G-ratio, was similar between wild-type and *Tuba1a* ^*ND/+*^ brains (Figure 4D; *p* = 0.34), as was axon diameter (Figure 4E; *p* = 0.14). There was a trend towards decreased myelination in *Tuba1a* ^*ND/+*^ animals (*p* = 0.07), but this difference was not statistically significant (Figure 4F). These data provide evidence that *Tuba1a* ^*ND/+*^ callosal axons do not correctly organize to form a commissure. Previously published data indicated that reduced developmental Tuba1a function is tolerable for cortical neuron migration (Gartz Hanson et al., 2016); however, our results indicate that reduced Tuba1a is not sufficient to support commissure formation.

### *Tuba1a* is Necessary for Neurite Extension and Cytoskeletal Organization in Growth Cone

**FIGURE 5:**
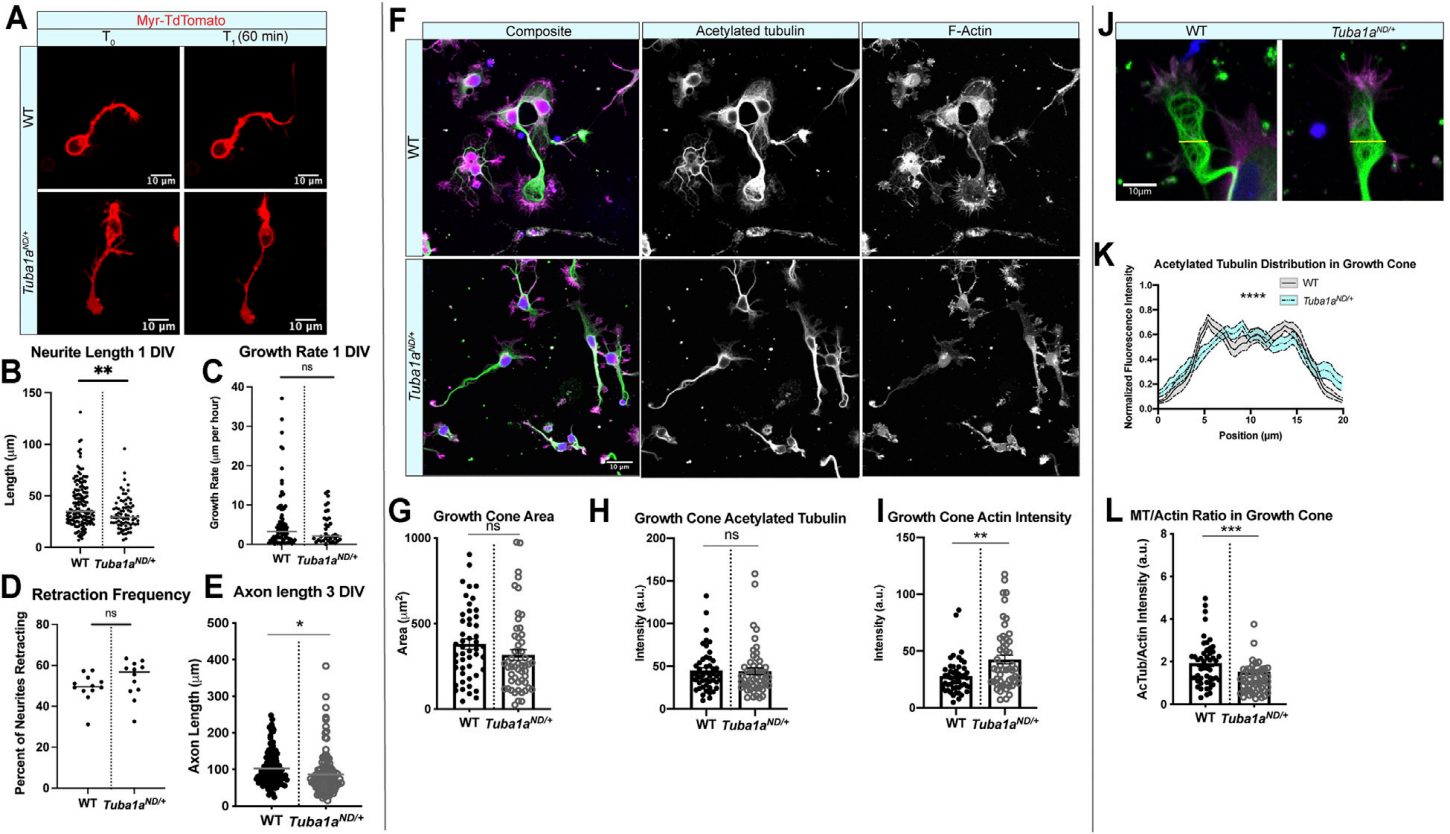
*Tuba1a* ^*ND*^ impairs neurite outgrowth and alters growth cone cytoskeleton in cortical neurons. **(A)** Representative time-lapse images of membrane-labeled Myr-TdTomato neurons at DIV 1. Myr-TdTomato images were acquired 60 min apart to assess neuronal growth rate in wild-type (WT; top) and *Tuba1a* ^*ND/+*^ (bottom) cortical neurons. **(B)** Neurite lengths from WT and *Tuba1a* ^*ND/+*^ mice at DIV 1. Data points represent individual neurons (32.51 ± 1.75 vs. 41.25 ±1.71 μm, *p* = 0.0016 by *t*-test, *n* = 157 WT neurons N = 13 mice and 77 Tuba1aND/+ neurons, N = 9 mice). **(C)** Scatter plot of neurite growth rate in DIV 1 neurons measured over 60 min using Myr-TdTomato tagged primary cortical neurons from WT and *Tuba1a* ^*ND/+*^ mice (5.828 vs. 4.064 μm/h, *p* = 0.1853). **(D)** Scatter plot of neurite retraction frequency reveals a trend toward more frequent retractions in *Tuba1a* ^*ND/+*^ mice (49.25 vs. 53.39%, *p* = 0.2247). **(E)** Scatter plot of neurite length at DIV 3 in fixed WT and *Tuba1a* ^*ND/+*^ primary cortical neurons. For each cell, the longest neurite, designated a putative “axon”, was measured from the cell soma to the distal neurite tip using an acetylated tubulin marker. Data points represent individual neurons (N = 3 mice, *n* = 124 neurons; *p* = 0.02). **(F)** Images of DIV 3 WT (top) and *Tuba1a* ^*ND/+*^ (bottom) cortical neurons stained with antibodies directed against acetylated tubulin (green) and rhodamine phalloidin (F-actin, magenta). Composite images are shown (left) with single channel grayscale images for acetylated tubulin (middle) and F-actin (right). Fluorescent images were post-processed in ImageJ to enhance brightness and contrast, set to a minimum value of 0 and a maximum value of 70. Scale bars are 10 µm. **(G)** Scatter plot of growth cone area at DIV 3 in WT and *Tuba1a* ^*ND/+*^ cortical neurons. Data points represent individual growth cones (N = 3 mice, *n* = 52 growth cones; *p* = 0.26). **(H)** Scatter plot of acetylated tubulin intensity within the growth cone of WT and *Tuba1a* ^*ND/+*^ cortical neurons at DIV 3. Acetylated tubulin images were collected using identical microscope imaging settings between samples, fluorescence of quantified images was not adjusted prior to measurement. Acetylated tubulin intensity was normalized to the area of the growth cone. Data points represent individual growth cones (*n* = 49 growth cones; *p* = 0.89). **(I)** F-actin intensity within growth cone of WT and *Tuba1a* ^*ND/+*^ cortical neurons at DIV 3. F-actin images were collected using identical microscope imaging settings between samples, fluorescence of quantified images was not adjusted prior to measurement. F-actin intensity was normalized to the area of the growth cone. Data points represent individual growth cones (N = 3 mice, *n* = 49 growth cones; *p* = 0.0014). **(J)** Representative images of WT and *Tuba1a* ^*ND/+*^ cortical neuron growth cones showing distribution of acetylated tubulin (green) and F-actin (magenta). The yellow line indicates where microtubule fluorescence intensity measurements were taken. **(K)** Line plot showing the average acetylated tubulin fluorescence intensity across a 20 µm line scan through the central domain of the growth cone, taken perpendicular to the extending neurite shaft or putative axon in DIV 3 WT and *Tuba1a* ^*ND/+*^ neurons. Differences between WT and *Tuba1a* ^*ND/+*^ growth cones were assessed by two-way ANOVA which showed a significant interaction between genotype and fluorescence intensity by position (*n* = 20 growth cones; *p* < 0.0001). **(L)**. Scatter plot representing the ratio of acetylated tubulin to F-actin intensity within the growth cones of DIV 3 WT and *Tuba1a* ^*ND/+*^ cortical neurons. Acetylated tubulin and F-actin intensity was measured from a single ROI in each growth cone, fluorescent images were acquired using the same microscope settings between samples and were not post-processed to adjust fluorescence. Data points represent individual growth cones (N = 3 mice, *n* = 49 growth cones; *p* = 0.0003 by Mann-Whitney test). For all plots, lines represent mean and error bars report SEM. Differences between WT and *Tuba1a* ^*ND/+*^ datasets were assessed by *t* test unless otherwise noted. *p < 0.05; **p < 0.01; ***p < 0.001; ****p < 0.0001.

To assess potential mechanisms by which reduced Tuba1a prevents commissural axons from crossing the midline, we measured neurite lengths, growth rates, and retraction frequency in cultured primary cortical neurons from P0 wild-type and *Tuba1a* ^*ND/+*^ mice (Figure 5A). *Tuba1a* ^*ND/+*^ neurites were significantly shorter than wild-type neurites (32.51 ±1.75 vs. 41.25 ±1.71 μm, *p* = 0.0016 by *t*-test, *n* = 157 WT neurons N = 13 mice and 77 *Tuba1a* ^*ND/+*^ neurons, N = 9 mice) at DIV1 (Figure 5B). *Tuba1a* ^*ND/+*^ neurites trended towards slower growth rates (5.828 vs. 4.064 μm/h, *p* = 0.1853 by *t*-test, Figure 5C) and increased frequency of retraction (49.25 vs. 53.39%, *p* = 0.2247 by *t*-test, Figure 5D) in an hour. Measurements of the longest neurite, the putative “axon”, at DIV 3 revealed that *Tuba1a* ^*ND/+*^ neurites remained significantly shorter than those of wild-type neurons (Figure 5E; N = 3 mice, *n* = 124 neurons; *p* = 0.02 by *t*-test). Together, these data show that developing neurons with reduced Tuba1a have a diminished capacity for neurite growth compared to wild-type neurons.

To determine if there are differences in the cytoskeleton network that explain shorter *Tuba1a* ^*ND/+*^ neurites, we assessed the abundance of acetylated microtubules and filamentous actin (F-actin) in developing growth cones of wild-type and *Tuba1a* ^*ND/+*^ cortical neurons at DIV 3 (Figure 5F). The growth cone is a dynamic developmental structure that uses the coordinated action of the actin and microtubule cytoskeleton to drive neuronal outgrowth in response to internal and external cues (Dent et al., 2011; Craig, 2018). The area of *Tuba1a* ^*ND/+*^ growth cones trended towards reduced area (317.4 ± 31.3 µm^2^) compared to wild-type (380.9 ± 30.4 µm^2^; Figure 5G; *n* = 49 growth cones; *p* = 0.15, by student’s t-test). We examined the amount of acetylated tubulin, a post-translational modification associated with stable microtubules (Figure 5H) and found there to be no difference in the overall fluorescence intensity of acetylated tubulin in *Tuba1a* ^*ND/+*^ neurons compared to wild-type (*n* = 49 growth cones; *p* = 0.89). In contrast, we observed a significant increase in F-actin fluorescence intensity within the growth cones of *Tuba1a* ^*ND/+*^ neurons compared to wild-type (Figure 5I; *n* = 49 growth cones; *p* = 0.0014). Normally, neuronal microtubules splay out in the central, actin-dominated regions of the growth cone, but are bundled towards the peripheral domains of the growth cone (Buck and Zheng, 2002). To assess the degree of growth cone microtubule bundling, we next performed line scans across the widest point of DIV 3 growth cones ≥10 µm (Figure 5J), an assay that was modeled after similar experiments in Biswas and Kalil (2018) (Biswas and Kalil, 2018). Line scans of acetylated tubulin through the growth cone revealed differences in microtubule organization between wild-type and *Tuba1a* ^*ND/+*^ neurons (Figure 5K; *n* = 39 growth cones; *p* < 0.0001 between genotypes by two-way ANOVA). Specifically, we observed peaks in fluorescence, indicating bundled microtubules, at the edges of the growth cone in wild-type neurons, where acetylated tubulin was more diffuse and lacked obvious organization in *Tuba1a* ^*ND/+*^ growth cones (Figure 5K). The ratio of acetylated microtubules to F-actin in the growth cone was significantly reduced in *Tuba1a* ^*ND/+*^ neurons compared to wild-type, indicating changes to the overall growth cone cytoskeletal environment in *Tuba1a* ^*ND/+*^ neurons (Figure 5L; *n* = 49 growth cones per genotype; *p* = 0.0003). Together, these data indicate that neurite growth is particularly sensitive to the amount of Tuba1a tubulin available, and that reduction in Tuba1a leads to shorter neurites (a result of subtle changes to growth and retraction rates) with growth cones containing abnormal actin and microtubule architecture.

### *Tuba1a* ^*ND/+*^ Neurites Exhibit Short Term Growth Increase With Global Addition of Netrin-1

**FIGURE 6:**
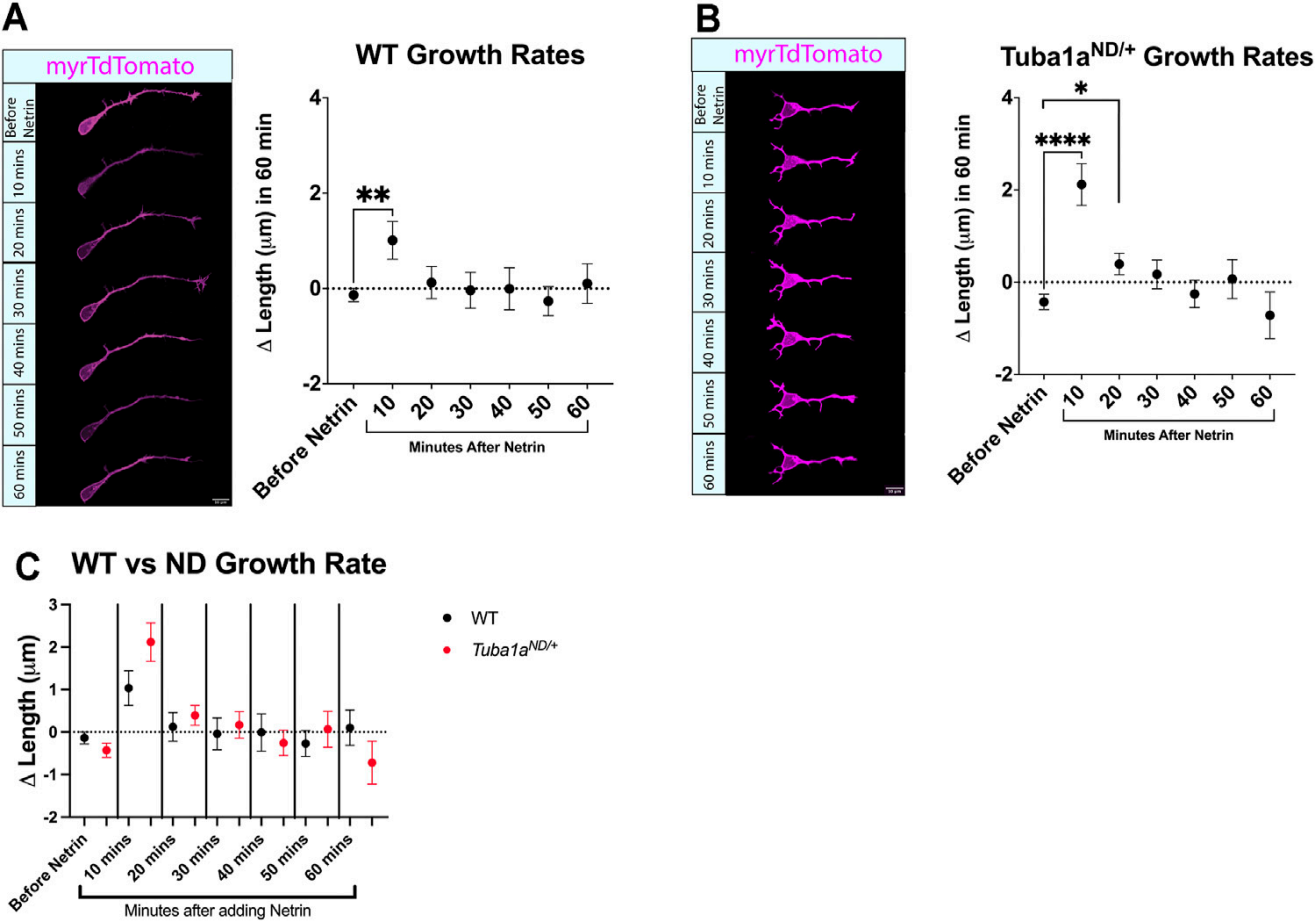
Reduced Tuba1a does not interfere with netrin-1-induced axon growth rates. **(A)** Primary cortical neuron cell cultures were made from *Tuba1a* ^*ND/+*^ mice and wildtype littermates. Cells were plated on glass-bottom dishes and images were collected every 10 min for an hour. Purified Netrin-1 was then added to the dish at 500 ng/ml and images were taken every 10 min for an additional hour. (*n* = 77 neurons; *p* = 0.0057, unpaired *t*-test) **(B)**. Analysis was repeated for *Tuba1a* ^*ND/+*^ mice (*n* = 91 neurons; *p* < 0.0001, and *n* = 83 neurons; *p* = 0.0488, unpaired *t*-test) **(C)**. To determine if there was a difference in stimulated growth between the wildtype and *Tuba1a* ^*ND/+*^ mice the growth values were compared at each time point. No differences were found at any time point.

Axon extension to form the corpus callosum is directed by guidance cues, including Netrin-1 (Nishikimi et al., 2013). Netrin-1 can act as an attractive guidance cue for developing axons by promoting directional axonal growth (Boyer and Gupton, 2018), and Netrin-1 deficient mice exhibit defects in commissural axon projection (Serafini et al., 1996) similar to that seen in *Tuba1a* ^*ND/+*^ mice (Figure 3). To determine if *Tuba1a* ^*ND/+*^ neurons respond to Netrin-1, primary cortical neurons were transfected with a membrane bound tdTomato reporter and cultured for 24 h. At DIV1, time lapse images were taken every 10 min over the course of an hour, then purified Netrin-1 was added to the media. Time lapse images taken after the addition of Netrin-1 revealed that both wild-type and *Tuba1a* ^*ND/+*^ neurons have a slight but significant increased growth rate at 10 min after the addition of Netrin-1 (Figures 6A,B). No differences were observed when comparing wild-type and *Tuba1a* ^*ND/+*^ neurons directly at the same time points (Figure 6C). These data indicate that both wild-type and *Tuba1a* ^*ND/+*^ neurons exhibit similar short-term responses to global addition of Netrin-1 at DIV1 in culture.

### *Tuba1a* ^*ND*^ Neurons Fail to Localize Critical Developmental Protein Map1b to Growth Cone

**FIGURE 7:**
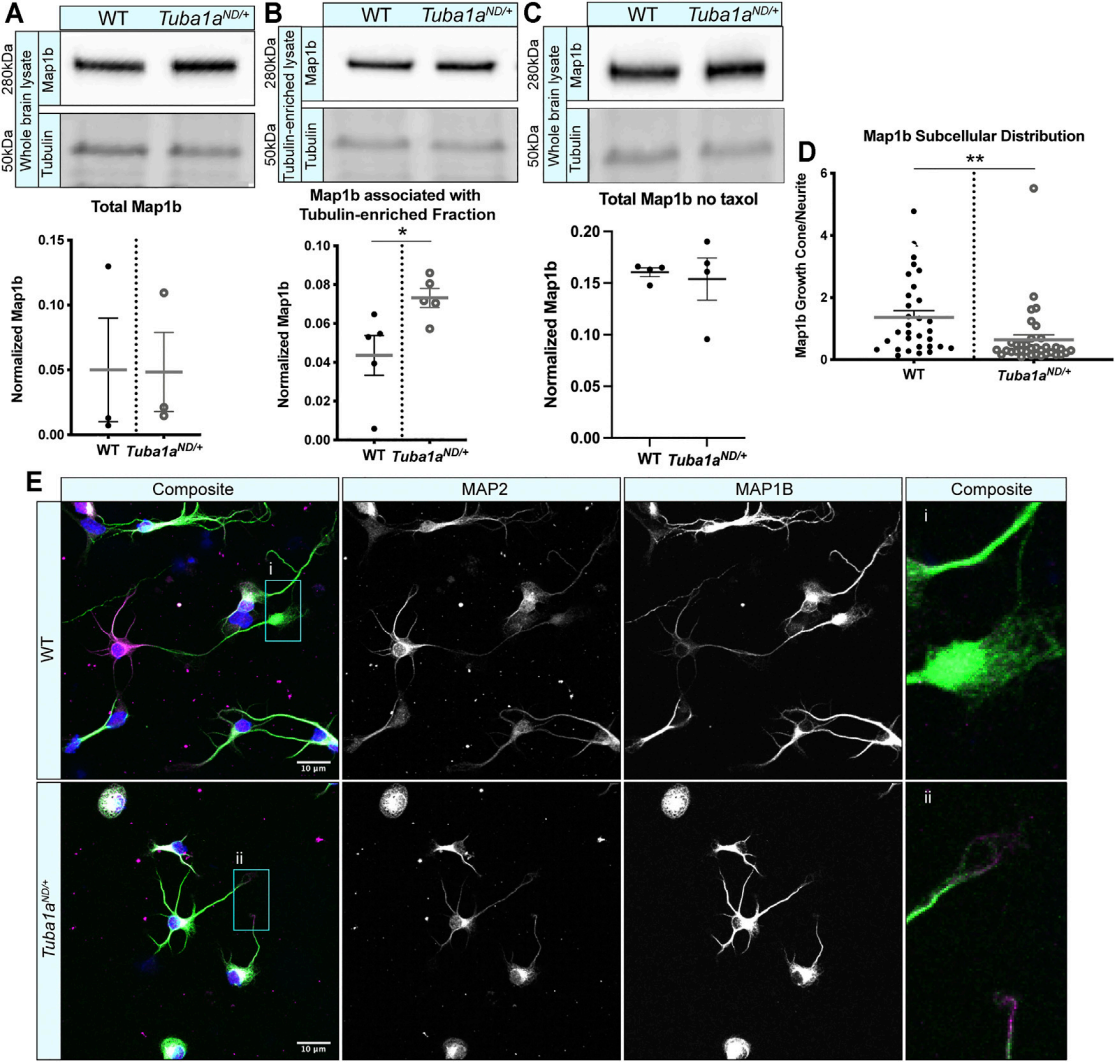
*Tuba1a* ^*ND*^ neurons do not correctly localize Map1b to the developing growth cone. **(A)** Western blot showing total Map1b protein in whole brain lysate from wild-type (WT) and *Tuba1a* ^*ND/+*^ mice. Scatter plot representing total Map1b protein in brain lysate by western blot, normalized to the total protein on a stain-free blot. *p* = 0.98 by *t*-test. **(B)** Western blots showing Map1b protein associated with a taxol stabilized tubulin-enriched fraction from brain (top panel). Due to the amount of protein that was loaded for Map1b western blots, antibody-stained bands for α-tubulin were oversaturated and could not be quantified, thus Map1b was normalized to the 50 kDa band (presumed to be primarily tubulin) on a UV-activated stain-free blot (bottom panel). Scatter plot quantifying Map1b associated with the taxol-stabilized tubulin-enriched brain lysate, normalized to the 50 kDa presumed tubulin band using stain-free western blotting. *p* = 0.03 by *t*-test **(C)**. Western blot showing Map1b from WT and *Tuba1a* ^*ND/+*^ pellet fraction of brain lysates without the addition of taxol. Scatter plot quantifying Map1b associated with tubulin enriched from brain lysates shows no significant difference between the amount of Map1b associated with tubulin when no taxol was added, *p* = 0.76 by *t*-test. Map1b is normalized to the 50 kDa tubulin band using stain-free western blotting. **(D)** Scatter plot showing the subcellular distribution of Map1b protein in WT and *Tuba1a* ^*ND/+*^ cortical neurons at DIV 3. Data are represented as Map1b fluorescent signal in growth cone region divided by a region proximal to the cell body of the same area. Fluorescent images were acquired using identical microscope imaging settings between samples and were not post-processed to adjust fluorescence prior to quantification. *p* = 0.009. **(E)** Representative images showing altered subcellular distribution of Map1b in *Tuba1a* ^*ND/+*^ (bottom) cortical neurons compared to WT (top) at DIV 3. Composite and individual channel grayscale images of MAP2 and Map1b immunocytochemistry are shown, *i* and *ii* indicate enlarged regions shown in insets. Scale bars are 10 m. Differences between groups were evaluated by *t* test. *p < 0.05; **p < 0.01.

MAPs play a crucial role in regulating neuronal microtubule function to support proper neurodevelopment. Microtubule-associated protein 1b (Map1b) regulates microtubule dynamics and facilitates actin/microtubule crosstalk to promote axon extension (Del Río et al., 2004). Mice lacking Map1b display an absence of the corpus callosum (Meixner et al., 2000; Gonzalez-Billault et al., 2001), similar to what is seen in *Tuba1a* ^*ND/+*^ mice. To assess whether *Tuba1a* ^*ND*^ might disrupt Map1b function, we performed a series of biochemical and immunocytochemical analyses. Map1b is highly enriched in neurons compared to other cells in the brain, and neuronal expression of Map1b accounts for most of the Map1b in the whole brain (Zhang et al., 2014; Zhang et al., 2016). Western blot analysis of whole brain lysates from wild-type and *Tuba1a* ^*ND/+*^ mouse brains showed no difference in the total amount of Map1b protein (Figure 7A; *p* = 0.98). There was no significant deficit in the association of Map1b with taxol-stabilized microtubules from *Tuba1a* ^*ND/+*^ brain lysates compared to wild-type; in fact, *Tuba1a* ^*ND/+*^ samples contained slightly more Map1b than wild-type (Figure 7B; *p* = 0.03). In addition, we performed a western blot to quantify and compare the amount of Map1b associated with polymerized tubulin in brains from WT and *Tuba1a* ^*ND/+*^ brains without taxol, at 37°C to avoid stimulating tubulin polymerization. We found that there was no difference in Map1b in the polymerized tubulin fraction (Figure 7C). These data indicate *Tuba1a* ^*ND*^ does not impair the interaction between Map1b and microtubules. In developing wild-type neurons, Map1b localizes strongly to the growth cone to promote axon growth and facilitate microtubule response to guidance cues (Figure 7D) (Black et al., 1994; Mack et al., 2000; Gonzalez-Billault et al., 2001; González-Billault et al., 2002; Tymanskyj et al., 2012). Strikingly, while *Tuba1a* ^*ND/+*^ neurons contained Map1b protein, they exhibited very little Map1b fluorescence in the growth cone compared to wild-type neurons (Figure 7E; *n* = 31 growth cones; *p* = 0.009). These data provide evidence that while the abundance of Map1b protein is unchanged by *Tuba1a* ^*ND*^, reduced Tuba1a dramatically impedes the subcellular localization of Map1b to the growth cone. Failure of *Tuba1a* ^*ND/+*^ neurons to localize Map1b to the developing growth cone provides a putative mechanism by which developing axons may fail to correctly organize the growth cone cytoskeleton, leading to the commissural deficits observed in *Tuba1a* ^*ND/+*^ mice.

## Discussion

### Functions of TUBA1A During Neurodevelopment

**FIGURE 8:**
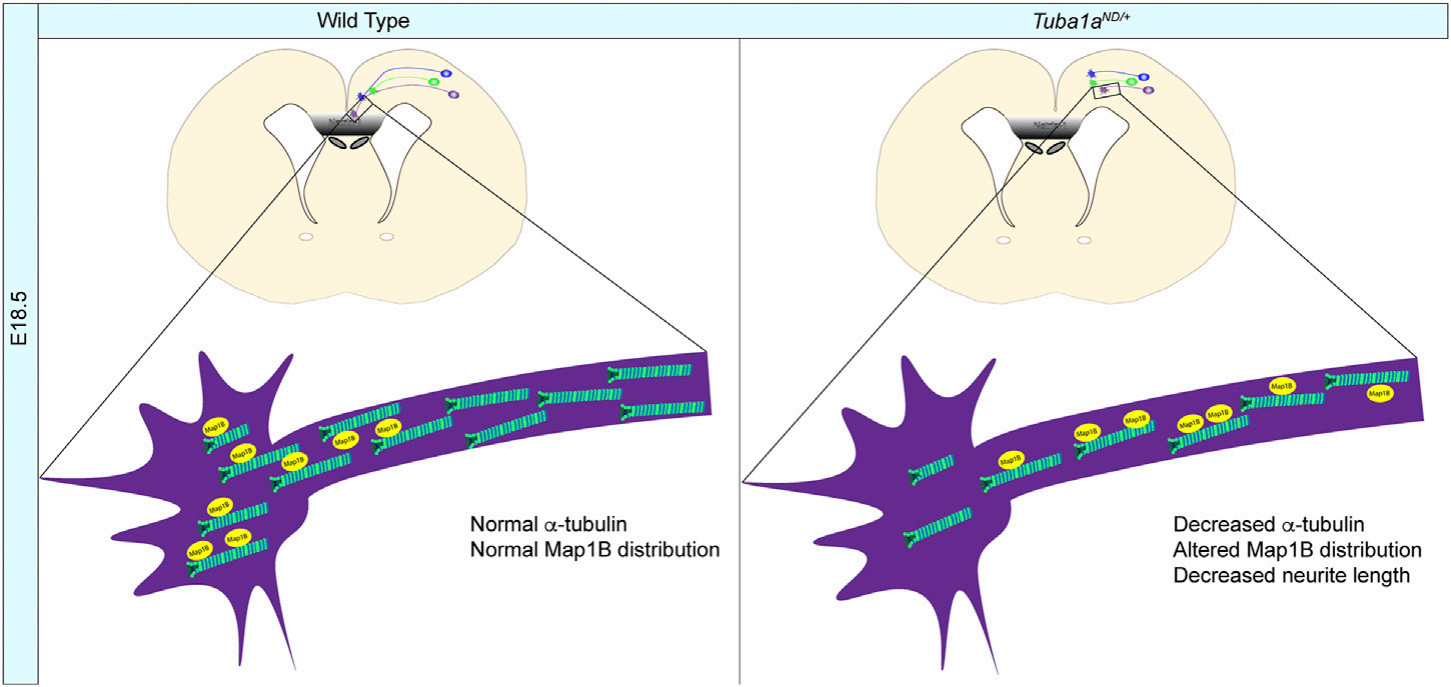
Mechanisms of Tuba1a-induced axonal pathfinding deficits. **(A)** Schematic illustrating how reduced Tuba1a may impair ability of *Tuba1a* ^*ND/+*^ axons to reach key signaling points at the correct developmental time to support proper brain formation. Wild-type axons (WT; left) reaching the midline crossing point of corpus callosum by embryonic day (E) 18.5 are compared to the potentially stunted axonal growth in *Tuba1a* ^*ND/+*^ brains (right). The reduced density of microtubules impair localization of Map1b and potentially other MAPs to contribute to slower growth of developing *Tuba1a* ^*ND/+*^ commissural neurons.

Using *TUBA1A*-His6 as a novel imaging tool, we illustrate that the *TUBA1A* ^*ND*^ allele is excluded from microtubule filaments and decreases the abundance of TUBA1A in cells (Figures 1, 2). We further demonstrate that mice with diminished Tuba1a exhibit normal cortical layering but are incapable of forming axon commissures. On a cellular level, decreased Tuba1a impairs neurite outgrowth (Figures 5–7) and disrupts growth cone localization of Map1b, which acts as a critical regulator of cytoskeletal dynamics during axonal outgrowth (Figure 8). The timing of axonal extension is precisely regulated, and growth cones which fail to reach targets at the correct time can miss crucial developmental signaling events. Interactions between MAPs and microtubules play a major role in adapting microtubule function in response to changing intra- and extra-cellular developmental environments (Meixner et al., 2000; Del Río et al., 2004; Tymanskyj et al., 2012). Therefore, it’s possible that neurons with reduced Tuba1a may be incapable of maintaining appropriate axonal growth rates or mounting appropriate cytoskeletal responses to extracellular guidance cues due to lack of localization of critical cargos such as MAP1B (Figure 8B). Our evidence supports a model in which adequate TUBA1A levels are required for neurite extension and commissure formation, which is achieved via a dynamic cytoskeleton that can readily respond to external cues through the action of critical factors localized to the growth cone. Without sufficient Tuba1a, axons cannot grow at the appropriate rate or correctly localize important MAP(s), rendering Tuba1a deficient axons incapable of crossing the midline to reach distant targets.

### Studying α-Tubulin Isotypes

*TUBA1A* has long been associated with neurodevelopment due to its spatial and temporal expression as well as its causal role in tubulinopathies (Miller et al., 1987; Gloster et al., 1994; Bamji and Miller, 1996; Gloster et al., 1999; Poirier et al., 2007; Fallet-Bianco et al., 2008; Kumar et al., 2010; Lecourtois et al., 2010; Oegema et al., 2015; Aiken et al., 2017), but cellular visualization of a single α-tubulin isotype or mutant has remained elusive due to the limited availability of isotype-specific tools. Studying individual α-tubulin isotypes in neurons has been historically arduous as the high degree of amino acid sequence similarity between α-tubulin isotypes (TUBA1A and TUBA1B proteins are 99.5% identical) leads to antibody promiscuity and has made genetically targeting a single α-tubulin gene challenging. The abundance of clinically identified mutations to *TUBA1A* provide strong evidence that *TUBA1A* is a major player in both tubulinopathy and typical neurodevelopment; however, the lack of available tools to study TUBA1A in cells has prevented researchers from understanding precisely how *TUBA1A* contributes to neurodevelopment. As such, previous studies of tubulinopathy mutations have relied heavily on mRNA analysis and indirect methods of evaluating TUBA1A protein function. Here we harness a previously-identified internal loop within TUBA1A that tolerates addition of small epitope tags without impacting TUBA1A incorporation or dynamics (Schatz et al., 1987; Heilemann et al., 2008; Hotta et al., 2016) to generate an important tool for the study of tubulinopathies and neuronal α-tubulin. Advantages of this tool are many. In Cos-7 cells and neurons, wild-type TUBA1A-His6 was able to incorporate into microtubule polymers, unlike TUBA1A containing a GFP fusion that prohibited incorporation into neuronal microtubules (Figure 2). Further, internal tagging does not interfere with C-terminal post translational modifications, which are abundant in neurons and contribute to function. The His6 tag can also be eluted natively so that the same tubulin isotype mutants can be purified for *in vitro* studies to complement localization and functional studies completed in cells. Additionally, the addition of the internal His6 tag did not remove or replace the Lysine 40 acetylation site*.* In Cos-7 cells and neurons, wild-type *TUBA1A-His6* was able to incorporate into microtubule polymers, unlike *TUBA1A* containing a GFP fusion that prohibited incorporation into neuronal microtubules (Figure 2). Importantly, this epitope-tagged TUBA1A can be used to gain insight into how mutant tubulin behaves in cellular microtubule networks (Figures 1, 2). For example, we reveal that *TUBA1A* ^*T349E*^, a substitution analogous to a previously described mutation that prevents tubulin polymerization in yeast (Johnson et al., 2011), fails to polymerize into neuronal microtubules but does not interfere with endogenous microtubule networks, as revealed by intact acetylated microtubules (Figure 2C). In contrast, *TUBA1A* ^*E255A*^, a lethal α-tubulin stabilizing mutation discovered in yeast which prevents tubulin depolymerization (Anders and Botstein, 2001; Roostalu et al., 2020), is abundantly incorporated into microtubule polymers and increases acetylated tubulin staining compared to wild type (Figure 2C). This mutation leads to thickened microtubule-rich protrusions (Figure 2C). Overall, this tool enables researchers to gain valuable insight into the cellular function of specific α-tubulin isotypes and determine how mutations impact tubulin function, microtubule networks, and cellular morphology.

When applied to *TUBA1A* ^*N102D*^, the epitope-tagged TUBA1A visualization revealed that the N102D mutation leads to polymerization-incompetent tubulin similar to the T349E substitution. Ectopic expression of *TUBA1A*-His6 protein in cells revealed that TUBA1A^ND^ protein is excluded from microtubule filaments (Figures 1B, 2C) and is approximately half as abundant as wild-type (Figure 1D), despite similar amounts of mRNA expression in transfected cells (Figure 1E). Based on this evidence, *TUBA1A* ^*ND*^ substitution lowers the total amount of usable α-tubulin in the cell (Figure 1D). Newly synthesized α- and β-tubulin proteins enter a complex tubulin folding pathway, where they interact with cytosolic chaperonins and tubulin-binding cofactors to become folded and assembled into tubulin heterodimers (Lewis et al., 1997). We found *TUBA1A* ^*ND*^ protein to be diminished compared to wild-type, and the introduction of the ND substitution into the primary yeast α—tubulin, *Tub1*, (*Tub1* ^*ND*^) is synthetic lethal when combined with tubulin folding pathway mutants. These data reveal an increased reliance on tubulin assembly cofactors when the ND mutation is present, which may be retained in mammalian systems (Gartz Hanson et al., 2016).

### Tuba1a Influences the Microtubule Cytoskeleton in Cells

The microtubule cytoskeleton supports a wide range of different cellular functions in different cell types, ranging from facilitating chromosome segregation during mitosis to forming dynamic and motile structures such as the neuronal growth cone. Understanding how different cells use the same basic building blocks to create vastly different microtubule-based structures is a major question in microtubule biology. Many different mechanisms have been identified by which cells regulate microtubule network properties and overall function. In this study, we provide the first evidence that TUBA1A is essential for regulating neuronal microtubule function to support commissure formation (Figures 3, 4). *Tuba1a* ^*ND*^ neurons exhibit deficits in neurite extension, [Figures 5, 6; (Buscaglia, 2020)], and do not support growth cone localization of at least one critical developmental MAP necessary for commissural axon pathfinding, Map1b (Figure 6). Collectively, these data support the conclusion that reduced Tuba1a is sufficient for certain microtubule-dependent neurodevelopmental stages, such as cortical neuron migration (Gartz Hanson et al., 2016), but does not allow for sufficient microtubule function to properly localize proteins to the growth cone or to grow at the rate necessary to form commissures. Thus, axons extending to reach distant targets may be exquisitely sensitive to α-tubulin levels.

We previously showed that while there was an overall reduction in α-tubulin protein in brains of heterozygous *Tuba1a* ^*ND/+*^ mice compared to wild type, there was no significant difference in the overall ratio of post-translational modifications, normalized to total α-tubulin (Buscaglia, 2020). Here, we show that the *Tuba1a* ^*ND*^ mutation changes the distribution of acetylated microtubules subcellularly in the growth cone (Figure 5), likely reflecting altered microtubule organization (Figure 5). Acetylation is a microtubule PTM that is associated with stable microtubule populations and as such is sparse in dynamic structures like growth cones (Schulze et al., 1987; Robson and Burgoyne, 1989; Mansfield and Gordon-Weeks, 1991; Palazzo et al., 2003; Baas et al., 2016; Eshun-Wilson et al., 2019). However, microtubule acetylation can be induced in growth cones following contact with extracellular matrix proteins and promotes cortical neuron migration *in vivo* and suppresses axon branching *in vitro*, demonstrating a clear role for this PTM in development (Dan et al., 2018; Bergstrom et al., 2007; Creppe et al., 2009). Tubulin PTMs, like acetylation, impact MAP-binding affinity and function, providing a clear mechanism by which changing the PTM landscape of microtubules could alter neuronal microtubule function (Mansfield and Gordon-Weeks, 1991; Bonnet et al., 2001; Reed et al., 2006; Janke and Kneussel, 2010; Lacroix et al., 2010; Sirajuddin et al., 2014; Balabanian et al., 2017). Thus, any changes to the organization or distribution of acetylated microtubules in the growth cone could impact the ability of developing neurons to appropriately navigate their environment and establish correct synaptic targets.

*Tuba1a* ^*ND/+*^ growth cones showed a significant increase in F-actin fluorescence compared to wild-type, causing an overall shift in the growth cone microtubule-actin balance (Figure 5). This may be due to a shift in distribution of F-actin rather than an increase in levels of F-actin. It is well established that interplay between the actin and microtubule cytoskeleton drives growth cone movements in developing neurons (Dent and Kalil, 2001; Dent et al., 2011; Piper et al., 2015; Craig, 2018). Growth cone microtubule polymerization induces F-actin assembly, and coordination of actin and microtubules is regulated by interactions with MAPs to drive appropriate growth cone response (Rochlin et al., 1999; Szikora et al., 2017; Biswas and Kalil, 2018; Slater et al., 2019). As actin and microtubules are tightly regulated within the growth cone, it is reasonable to assume that mutations which disrupt microtubule function, like *Tuba1a* ^*ND*^, likely also impact the actin cytoskeleton of developing neurons. In *Tuba1a* ^*ND/+*^ neurons, the actin cytoskeleton may occupy increased growth cone territory as the result of microtubule deficiencies, but additional testing of actin-response in developing *Tuba1a* ^*ND/+*^ neurons is needed to assess whether the increase in growth cone actin has any functional consequences.

We show that *Tuba1a* ^*ND/+*^ neurons do not effectively localize at least one developmental MAP, Map1b, to the growth cone (Figure 7). We previously demonstrated that axonal transport is impaired in developing *Tuba1a* ^*ND/+*^ neurons (Buscaglia, 2020), thus reduced Tuba1a could lead to altered localization of developmental MAPs due to disrupted trafficking. Our data do not support the conclusion that Map1b expression or binding to microtubules is reduced. Instead, we favor a model in which trafficking is disrupted so that Map1b is not appropriately localized. Intracellular transport is a crucial function of neuronal microtubules throughout life, and microtubule-based transport is essential during neurodevelopment for distributing cellular components into burgeoning neurites (Hoogenraad and Bradke, 2009; Feltrin et al., 2012; Liu et al., 2012; Baraban et al., 2013; Dent and Baas, 2014; Leung et al., 2018; Miller and Suter, 2018). Correct localization of developmental MAPs, mRNAs and organelles are necessary for cytoskeletal response to extracellular guidance cues (Welshhans and Bassell, 2011; Feltrin et al., 2012; Qu et al., 2013; Pilaz et al., 2016; Leung et al., 2018). In particular, Map1b is required for commissure formation (Meixner et al., 2000) and homozygous Map1b knockout animals have similar phenotypes to *Tuba1a* ^*ND/+*^ mice [Figure 3 and (Meixner et al., 2000)]. Map1b is necessary for neuronal response to the guidance cue, Netrin1, a key player in commissural formation (Serafini et al., 1996; Barallobre et al., 2000; Finger et al., 2002; Lindwall et al., 2007; Fothergill et al., 2014; Arbeille and Bashaw, 2018). While our data show that *Tuba1a* ^*ND/+*^ neurites grow faster in response to global application of Netrin-1 similar to wild type neurons in the time frame we measured, we cannot rule out impairment of turning in response to a localized source of Netrin-1 or response to other guidance cues. With the exception of tubulin and actin, Map1b is the most abundant cytoskeletal protein in the growth cone and localizes asymmetrically to the growth cone (Mack et al., 2000; Igarashi, 2014). Our data suggests that if Map1b is not localized to the growth cone, it cannot perform its function in commissure formation. Map1b acts downstream of several important developmental signaling pathways to regulate function of both actin and microtubules within the growth cone, and dysregulation of this or other MAPs could therefore impact multiple cytoskeletal components (Mack et al., 2000; Noiges et al., 2002; Tymanskyj et al., 2012).

### Reduced Tuba1a Function does not Adequately Support Neurite Growth for Development

The timing of axon growth is crucial for the growth cone to receive and respond to the appropriate guidance cues (Rossi et al., 2017; Krol and Feng, 2018). We showed that neuronal microtubules with reduced Tuba1a do not support axonal growth to the same degree as wild-type microtubules, causing shorter neurite length (Figure 5). If neurons lacking appropriate levels of TUBA1A do not reach the correct location at the correct time, it is possible that neurons will fail to receive key developmental signals (Figure 8). How does reducing Tuba1a function impair axon extension? One possibility is that there is not enough α-tubulin in the neurite or axon to assemble and extend microtubules. Another, non-exclusive possibility is that the microtubule tracks formed in *Tuba1a* ^*ND*^ axons are not sufficient to support trafficking of microtubule stabilizing factors. Microtubule-based transport is essential during neurodevelopment (Hoogenraad and Bradke, 2009; Feltrin et al., 2012; Liu et al., 2012; Baraban et al., 2013; Dent and Baas, 2014; Leung et al., 2018; Miller and Suter, 2018), and we previously showed that intracellular transport is impaired in developing *Tuba1a* ^*ND/+*^ neurons (Buscaglia, 2020). Map1b is not appropriately localized to the growth cone (Figures 7, 8), suggesting that diminished Tuba1a function in neurons impacts the subcellular localization of Map1b, and potentially other MAPs, to the growth cone through impairments to intracellular trafficking (Figure 8).

Human neurodevelopmental disorders that impact microtubule function, such as tubulinopathies, demonstrate that microtubules are critical for neurodevelopment. Tubulinopathy patients exhibit severe, sometimes lethal, brain malformations that frequently impact multiple neurodevelopmental processes, including neuronal survival, migration, and axon extension (Fallet-Bianco et al., 2008; Tian et al., 2008; Keays et al., 2010; Kumar et al., 2010; Tian et al., 2010; Bamba et al., 2016; Belvindrah, 2017; Aiken et al., 2019a; Aiken et al., 2019b). The range of phenotypes exhibited by tubulinopathy patients have made it challenging for scientists to pinpoint specific aspects of neuronal function that are reliant on TUBA1A tubulin. We used *Tuba1a* ^*ND*^ as a tool to interrogate the requirement for Tuba1a in discrete aspects of neurodevelopment. Importantly, commissural abnormalities, such as agenesis of the corpus callosum, are commonly reported features in tubulinopathy patients (Poirier et al., 2007; Fallet-Bianco et al., 2008; Cushion et al., 2013; Oegema et al., 2015; Aiken et al., 2017). Cortical malformations and neuronal migration errors are also common features of *TUBA1A* tubulinopathies; however, it has thus far been unclear whether commissural deficits occur as a primary or secondary consequence of TUBA1A dysfunction. In this study, we provide evidence that axons deficient in Tuba1a fail to properly navigate to meet contralateral binding partners. These data demonstrate that TUBA1A is required for forebrain commissural formation, independent of its role in neuronal survival or migration.

## Data Availability

The raw data supporting the conclusion of this article will be made available by the authors, without undue reservation.

## Supplementary Material

Supplementary Figure S1 dSTORM imaging of TUBA1A-His6 signal reveals single microtubules. **(A)** Wide-field (top panels) and dSTORM super-resolution (lower panels and insets) microscopy of DIV7 neurons transfected with TUBA1A-His6 plasmid DNA and immunolabeled with α-His6 antibody. Yellow boxes reveal the location of the magnified insets below. **(B)** dSTORM super-resolution microscopy of DIV4 astrocytes immunolabeled with total α-tubulin antibody (left panels) or transfected with TUBA1A-His6 plasmid DNA and immunolabeled with α-His6 antibody. Yellow boxes reveal the location of the magnified insets below.
