# Supplementary figures and images for: Bridging the Gap: The Importance of TUBA1A α-Tubulin in Forming Midline Commissures
Source: Front Cell Dev Biol. 2022 Jan 19;9:789438. doi: 10.3389/fcell.2021.789438 (PMC8807549; doi:10.3389/fcell.2021.789438)

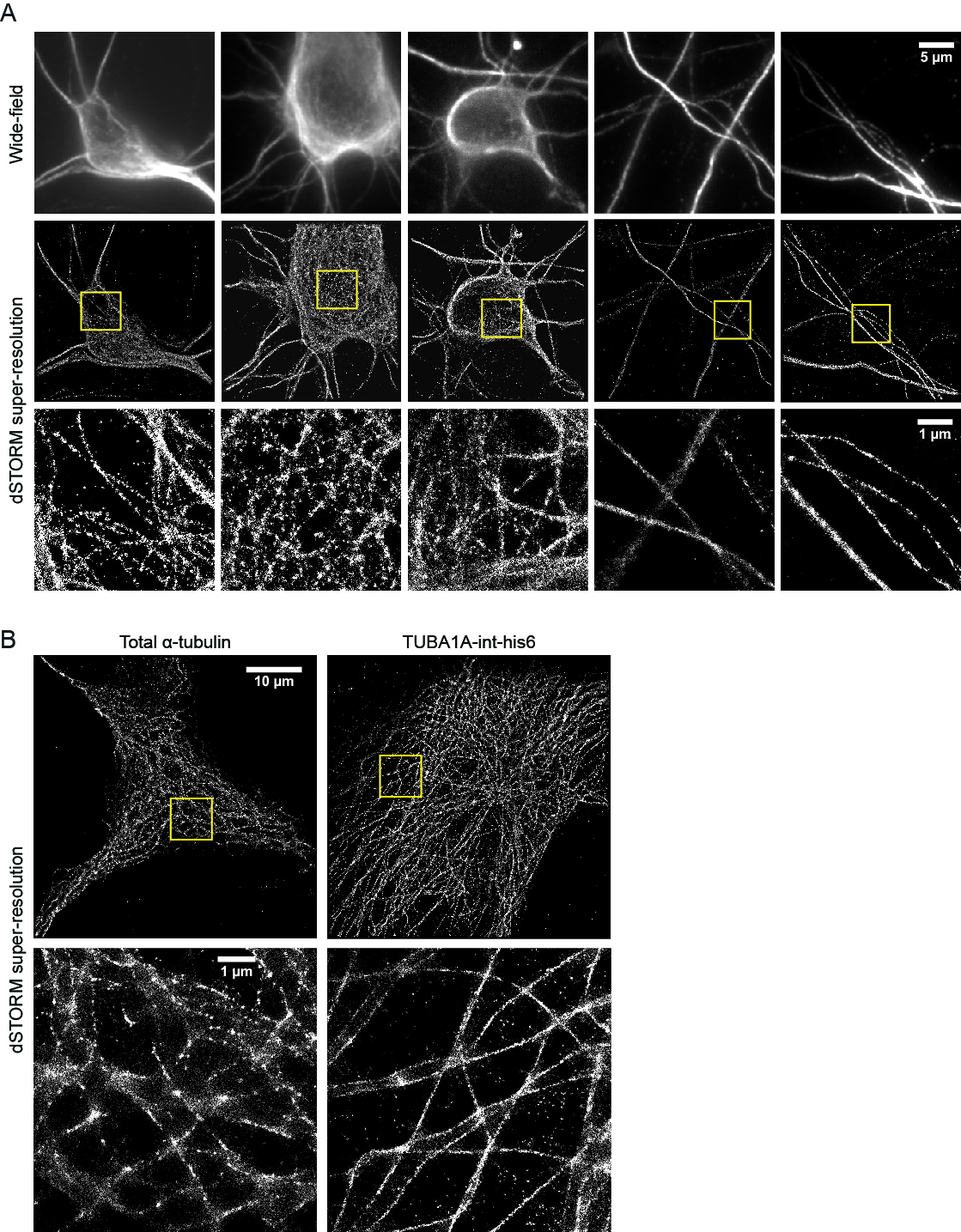

Supplement: Supplementary file 1 [file Image1.jpg]
